# Supplementary material for: Butyrophilin-2A1 Directly Binds Germline-Encoded Regions of the Vγ9Vδ2 TCR and Is Essential for Phosphoantigen Sensing
Source: Immunity. 2020 Mar 17;52(3):487–498.e6. doi: 10.1016/j.immuni.2020.02.014 (PMC7083227; doi:10.1016/j.immuni.2020.02.014)
Supplement: Document S1. Figures S1-S5 and Tables S1 and S2 [file mmc1.pdf]

Immunity, Volume *52*

Supplemental Information

Butyrophilin-2A1 Directly Binds Germline-Encoded Regions of the Vγ9Vδ2 TCR and Is Essential for Phosphoantigen Sensing

Mohindar M. Karunakaran, Carrie R. Willcox, Mahboob Salim, Daniel Paletta, Alina S. Fichtner, Angela Noll, Lisa Starick, Anna Nöhren, Charlotte R. Begley, Katie A. Berwick, Raphaël A.G. Chaleil, Vincent Pitard, Julie Déchanet-Merville, Paul A. Bates, Brigitte Kimmel, Timothy J. Knowles, Volker Kunzmann, Lutz Walter, Mark Jeeves, Fiyaz Mohammed, Benjamin E. Willcox, and Thomas Herrmann

Immunity, Volume *52*

Supplemental Information

Butyrophilin-2A1 Directly Binds Germline-Encoded Regions of the Vγ9Vδ2 TCR and Is Essential for Phosphoantigen Sensing

Mohindar M. Karunakaran, Carrie R. Willcox, Mahboob Salim, Daniel Paletta, Alina S. Fichtner, Angela Noll, Lisa Starick, Anna Nöhren, Charlotte R. Begley, Katie A. Berwick, Raphaël A.G. Chaleil, Vincent Pitard, Julie Dechanet-Merville, Paul A. Bates, Brigitte Kimmel, Timothy J. Knowles, Volker Kunzmann, Lutz Walter, Mark Jeeves, Fiyaz Mohammed, Benjamin E. Willcox, and Thomas Herrmann

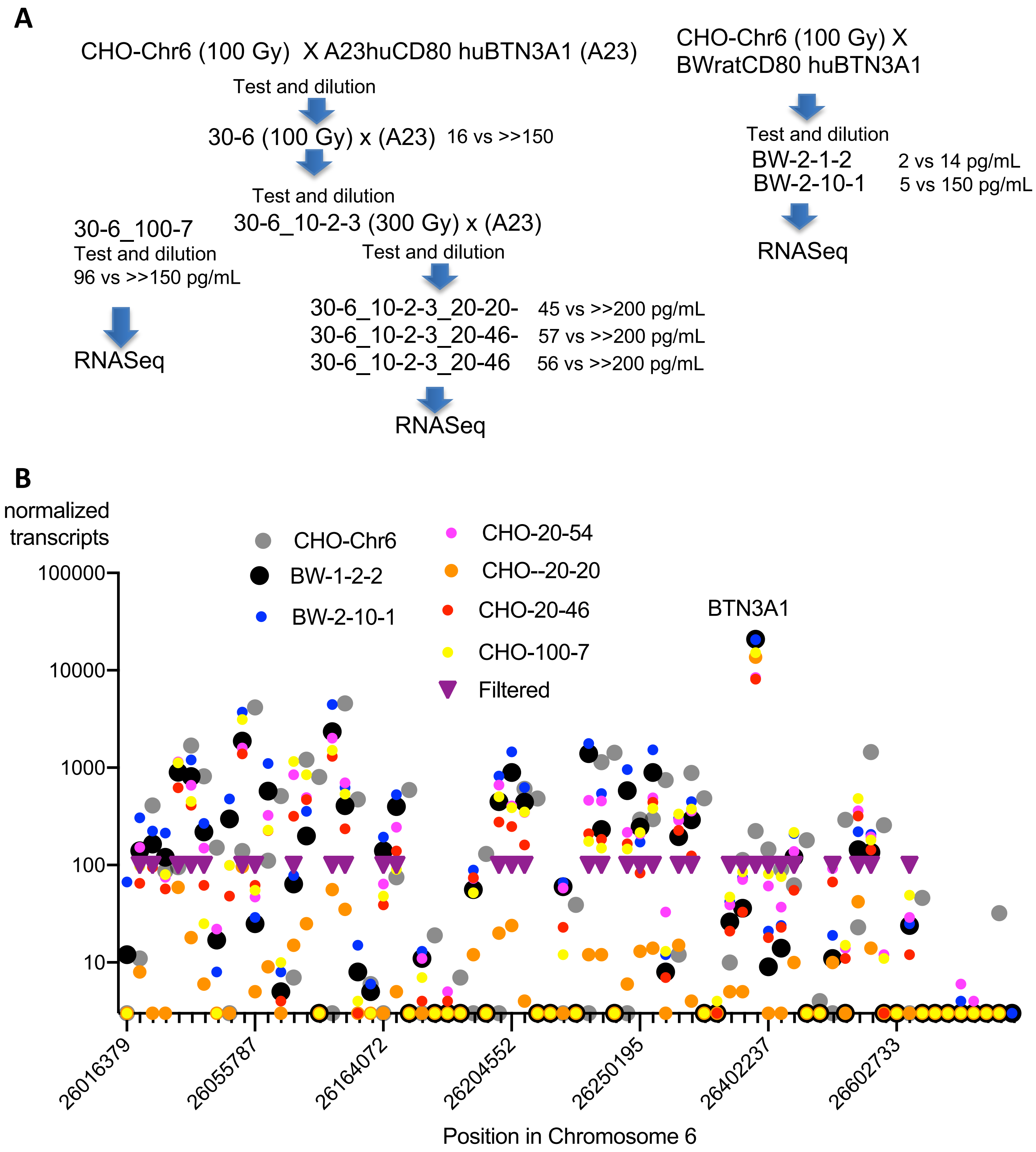

Supplementary Figure 1, related to Figure 1. Strategy used for identification of BTN2A1 as Factor X. (A) Origin of radiation hybrid clones selected for transcriptional analysis. IL-2 production (pg/ml) in the stimulation assay in the presence of medium or with 1 µM HMBPP. (B) Region of human Chr 6 implicated in potentiation of P-Ag sensitisation by rodent-human radiation hybrids. Each dot represents a gene detected as ≥3 transcripts, with those highlighted in pink detected in every P-Ag-sensitizeable clone selected for analysis.

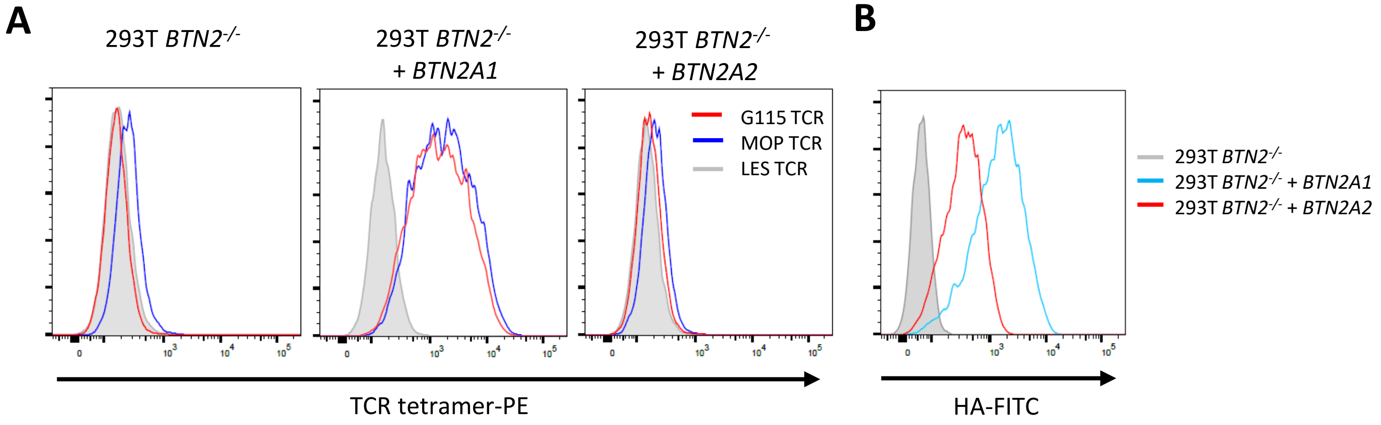

Supplementary Figure 2, related to Figure 2. Vγ9Vδ2 TCR tetramer stains BTN2A1 transductants. (A) 293T *BTN2*$^{-/-}$ cells transduced to express HA-tagged BTN2A1 and BTN2A2 were stained with the indicated TCR tetramers. (B) Flow cytometry analysis of HA-tagged BTN2A1 and BTN2A2 in 293T *BTN2*$^{-/-}$ cells, as detected by anti-HA mAb.

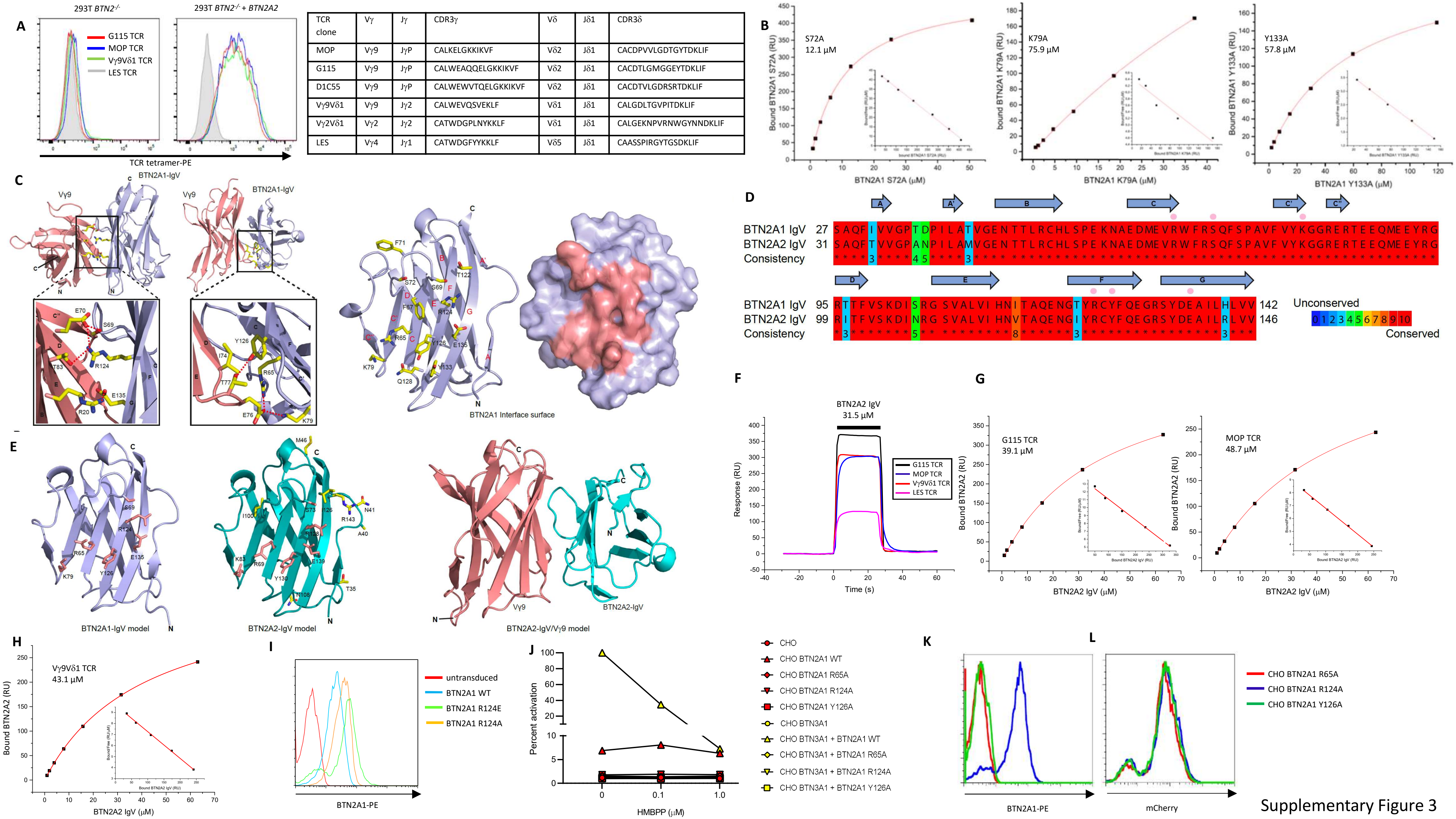

TCR clone	Vγ	Jγ	CDR3γ	Vδ	Jδ1	CDR3δ
MOP	Vγ9	JγP	CALKELGKKIKVF	Vδ2	Jδ1	CACDPVVLGDTGYTDKLIF
G115	Vγ9	JγP	CALWEAQQELGKKIKVF	Vδ2	Jδ1	CACDTLGMGGEYTDKLIF
D1C55	Vγ9	JγP	CALWEWVTQELGKKIKVF	Vδ2	Jδ1	CACDTVLGDRSRTDKLIF
Vγ9Vδ1	Vγ9	Jγ2	CALWEVQSVEKLF	Vδ1	Jδ1	CALGDLTGVPITDKLIF
Vγ2Vδ1	Vγ2	Jγ2	CATWDGPLNYKKLF	Vδ1	Jδ1	CALGEKNPVRNWGYNNDKLIF
LES	Vγ4	Jγ1	CATWDGFYYKKLF	Vδ5	Jδ1	CAASSPIRGYTGSDKLIF

Supplementary Figure 3

Supplementary Figure 3, related to Figure 3. BTN2A1 and BTN2A2 bind germline encoded regions of Vγ9. (A) (Left panel) Vγ9Vδ1-TCR tetramer staining of BTN2A1-transduced 293T *BTN2*$^{-/-}$ cells. (Right panel) Comparison of CDR3γ sequences in G115, MOP, D1C55, and Vγ9Vδ1 TCR clonotypes. (B) Equilibrium binding of BTN2A1 mutants S72A (left; 12.1 µM)), K79A (middle, 75.9 µM) and Y133A (right, 57.8 µM) to G115 TCR. Results are representative of two independent experiments. (C) (Left two panels) Vγ9/BTN2A1 interface from the mutagenesis-informed HADDOCK model of the interaction. (Right two panels) BTN2A1 interface residues (left) forming a surface (right) proposed to interact with Vγ9 IgV. (D) Comparison of BTN2A2 and BTN2A1 IgV sequence. Pink dots indicate conservation of BTN2A1 IgV amino acids predicted to be involved in binding Vγ9 IgV, in BTN2A2. (E) Homology model of BTN2A2 IgV, alone and in complex with Vγ9 TCR. (F) Specific binding of BTN2A2 IgV (31.5 µM) to Vγ9Vδ2 TCR as detected by SPR. (G) Equilibrium affinity and Scatchard (inset) analysis of BTN2A2 IgV binding to G115 (39.1 µM) and MOP (48.7 µM) Vγ9Vδ2 TCRs. (H) Equilibrium affinity analysis of BTN2A2 IgV binding to Vγ9Vδ1 TCR (43.1 µM). (I) Flow cytometry staining of BTN2A1 R124A and BTN2A1 R124E mutants at surface of BTN3A1-expressing CD80^{+} CHO cells. (J) Effects of BTN2A1 mutations on IL-2 production by TCR-MOP in response to HMBPP-treated BTN3A1 and BTN2A1 expressing CD80^{+} CHO cells. (K) Flow cytometry staining of BTN2A1 R65A, R124A and Y126A mutants at surface of BTN3A1-expressing CD80^{+} CHO cells. (L) mCherry reporter detection in BTN2A1 R65A, R124A and Y126A mutants-expressing CD80^{+} CHO cells.

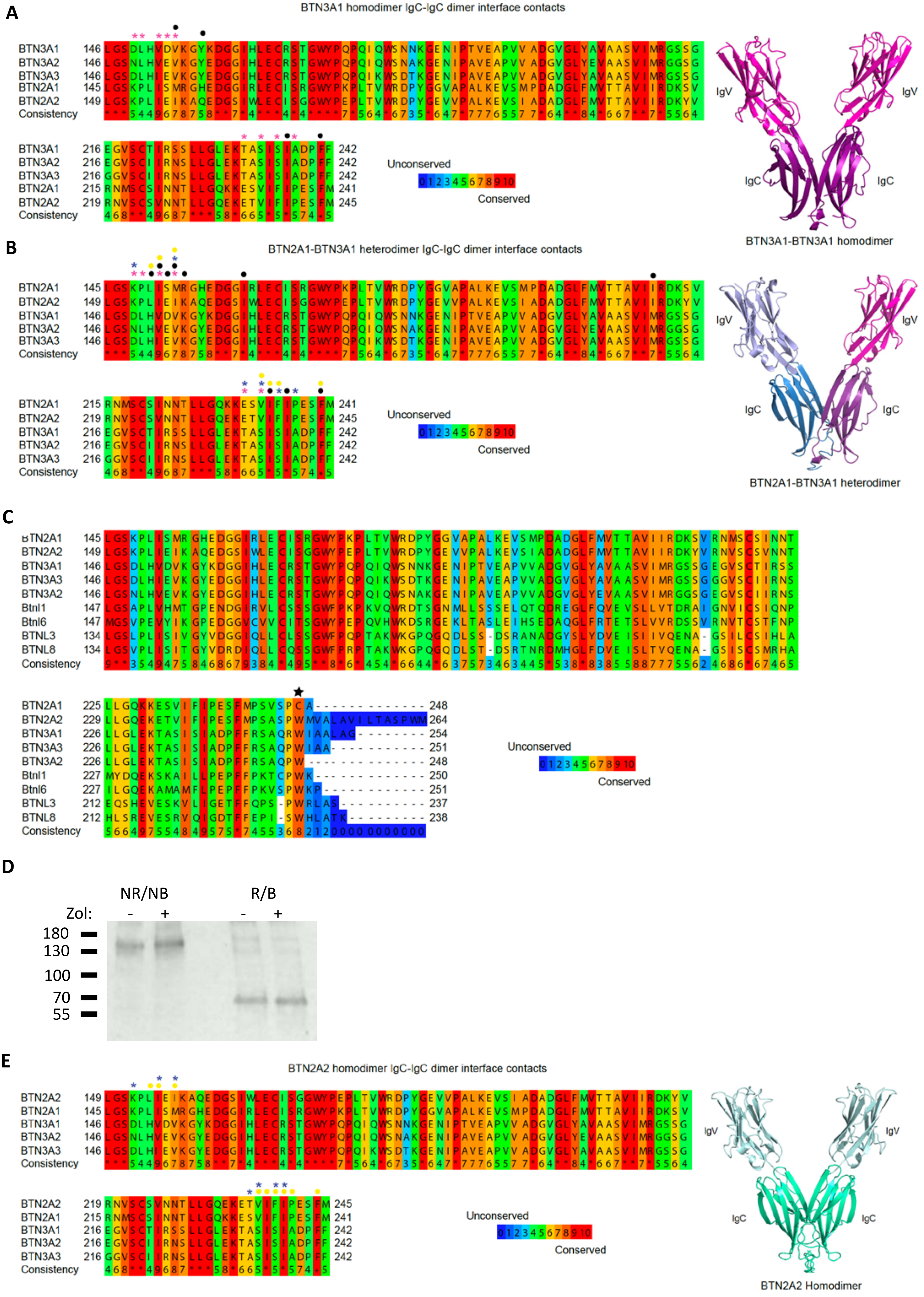

Supplementary Figure 4

Supplementary Figure 4, related to Figure 4. BTN2A1 can form a homodimer involving hydrophobic IgC-IgC contacts and a disulphide linkage. (A) Sequence alignment showing conservation of BTN3A1 IgC-IgC interface residues in BTN2A1. Pink stars indicate BTN3A1 hydrogen bonding interactions mediated by main chain atoms (D149, L150, V152, D153, V154, T232, S234, S236 and A238); black spheres indicate BTN3A1 hydrophobic interactions (V154, Y157, I237, and F241). (B) Sequence alignment showing potential for heterodimer interactions involving BTN2A1. Pink stars indicate BTN3A1 hydrogen bonding interactions mediated by main chain atoms (D149, L150, V152, V154, T232 and S234); black spheres indicate BTN3A1 hydrophobic interactions (H151, V152, D153, V154, K155, I162, M210, I235, I237 and F241); blue stars indicate BTN2A1 hydrogen bonding interactions mediated by main chain atoms (K148, M153, E231, V233, F235 and P237); yellow spheres indicate BTN2A1 hydrophobic interactions (L150, I151, M153, V233, I234, F235 and F240). (C) Sequence alignment showing unique membrane proximal Cys residue (highlighted by a black star) in BTN2A1 relative to other BTN/BTNL molecules. (D) BTN2A1 homodimer formation is not affected by Zol treatment. $CD80^+$ CHO cells expressing BTN2A1-HA and FLAG-BTN3A1 were treated +/- Zol (20 µM) overnight, surface biotinylated, then lysed and immunoprecipitated using anti-HA antibody, and blotted with streptavidin-horseradish peroxidase. (E) BTN2A2 dimer formation. Homology model of BTN2A2 indicating potential for IgC-IgC homodimers. Blue stars indicate BTN2A2 hydrogen bonding interactions mediated by main chain atoms (K152, I155, I157, T236, V237, F239 and I240); yellow spheres indicate BTN2A2 hydrophobic interactions (L154, I155, I157, V237, I238, F239, I240, P241 and F244).

Supplementary Figure 5

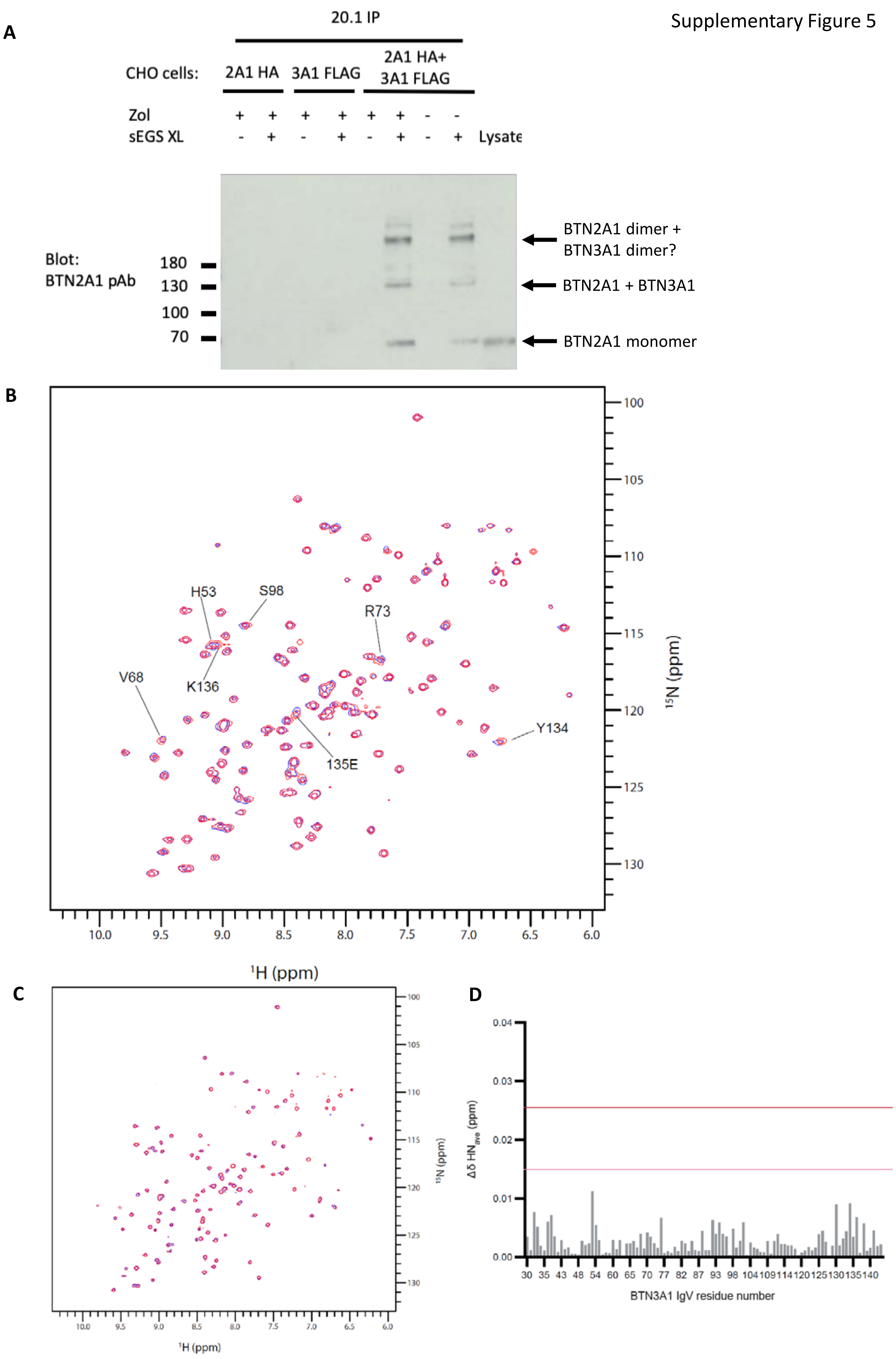

Supplementary Figure 5, related to Figure 5. BTN2A1 associates with BTN3A1 via IgV-IgV contacts. (A) SDS-PAGE following surface crosslinking and lysis of CHO CD80^{+} cells transduced with BTN2A1-HA and FLAG-BTN3A1, and subsequent immunoprecipitation using 20.1 anti-BTN3A1 mAb, followed by western blot using anti-BTN2A1 polyclonal antibody. (B) NMR chemical shift perturbation analysis of BTN3A1 IgV domain (100 μM) with BTN2A1 IgV. Overlay of ^{1}H-^{15}N HSQC spectra of ^{1}H-^{15}N-labelled BTN3A1 IgV domain in the absence (red) and presence (100 μM; blue) of BTN2A1 IgV domain. Labelled residues are those undergoing CSPs above the threshold set in Figure 5D. (C) NMR chemical shift perturbation analysis of BTN3A1 IgV domain (120 μM) with BTNL3 IgV. Overlay of ^{1}H-^{15}N HSQC spectra of ^{1}H-^{15}N-labelled BTN3A1 IgV domain in the absence (red) and presence (320 μM; blue) of BTNL3 IgV domain. (D) Graph of chemical shift versus residue number in BTN3A1 IgV following addition of BTNL3 IgV domain as in (C). Threshold levels for significant CSPs are indicated by horizontal lines as in Figure 5D.

Supplementary Table 1, related to main text Figure 1 and Key Resource Table. Primers and genomic markers used for characterization of radiation hybrids.

Marker	Fwd primer	Rev primer	Source	Identifier
Ly86	AAATCTTCTTCCCCTGGGAA	GAGGATGGCTGTTAGATCATCC	This Paper	N/A
CD83	TAAGGGAAAGCCAACAATGG	CACGGTCTGTTCTTGAAGCA	This Paper	N/A
RN7SL	GAATGTGGATGGGCTGTCTC	GCCTCAAACAGTGCATTTATACTT	This Paper	N/A
MYLIP	CAGACATGCAGAAAGTTGCA	ATGCACACAGAAGATAAAGCA	This Paper	N/A
NUP153	GGACTCTCAGGCAGGAAT	TGGAACAGCCCAATAGGT	This Paper	N/A
RPL21	CCGCGGAATTCTTAGTTATCCTCT	TTTTTGCAAAGCTCACACCTTCT	This Paper	N/A
RPL36	TGTGTAGCGGAAGACATCTAGCA	TCCATGAGCACTGGTAAGAATGA	This Paper	N/A
Prolactin	GGTTTCTGATACACTGGCCCGATAT	GGAACGGATCATTAAAGGACCTTCTC	Cathy Abbot	N/A
LOC1053	TCAGACCAAGAACAACGAGGAAC	ACTCAACACGTGTCACACACAGA	This Paper	N/A
ACOT13	GTATTGTAACAGGGGTCATGCA	CCCCAAGCTGAGACATGATT	This Paper	N/A
C6orf229	GATCCTGCTGCTCCAGAGTG	AAGCTCCTTGACGCAAGACA	This Paper	N/A
MTCO2P33	CAATTTGGCCTTCAAACGCT	AACACGTAAGGATAGGAGGGC	This Paper	N/A
RP3-425P12	AGATCATTTCCTGAAGGCCGA	CCTTGCATTCTTTGGCGGGT	This Paper	N/A
SLC17A4	AGGCTGAGGCATCCCTA	TGTACCCCGGCTAATAAGTT	This Paper	N/A
BTN3A2	AGCACTTTACTGATATTCATTC	AAAGTCAATACACTTTTCTACAC	This Paper	N/A
OR2B2	AATTTAAAATCACAGTGAGTAG	AGATGGAGGTTCAGATAG	This Paper	N/A
MOG	AACTGAAAGGGTTCCGTGTG	ATTTCTGACCATTTGCCCTG	This Paper	N/A
TNFA	CAGGGTCCTACACACAAATCAGTCA	AAGAGAACCTGCCTGGCAGCTTGT	This Paper	N/A
HLA-DQ	GGTTATCACTCTTCTGTGATGCC	TCTGGGTCAGTGCAGGAAG	This Paper	N/A
STK38	TTGCTACTCAGCCTATTACTTTT	AAGGTTATACAGGACGACATAAC	This Paper	N/A
NFKBIE	GCCTTTTACTTCAACTGCCCTCT	ACAACAGGTGCCAGTTACAGGTT	This Paper	N/A
ICK	ACAAAAAAGCCTACTGCATTCA	CTGTCTGCCTTGGTGATGAATC	This Paper	N/A
EEF1A1	CCAACCTGAGATGTCCCTGT	TGAGTCACCCACACAAAGGA	This Paper	N/A
MAP3K7	TTGCTTAACCTTTTCCATTTCC	TCTGAATGTCACAGGCTGTACC	This Paper	N/A
CD164	GAGGCACCCCATAACACTTG	TGAATTTTGGAGATGGAATGC	This Paper	N/A
PTPRK	CCACCATTATTCAATTCCAAGCAT	TGGTCTCTTAATGCCTGATTGGT	This Paper	N/A
MYB	ACAAGCATGCGTTGCCACTTCTT	CAAACACAGGATCCATGCAACA	This Paper	N/A
ULBP2	CATCAAAATGCGCATTCATC	CCAGATCAGAATGGGTGCTT	This Paper	N/A
ESR	CTCTCTCTCTGCGCATTCAG	GAAGCCCAGAGATGCCTCAC	Cathy and S	N/A
SCAF8	TGGCACTAAATGAGGAAAAA	TGCCTTCATGGAGTTTACAT	This Paper	N/A
MAS1	CGGTCACAGTTGAGACTGTCGTCT	TTAGTATCTCATGCATATGGGATGAG	Theune et a	N/A
SLC44A4	GTCCTCTCCCACAAAAAAGTCC	TCAGCCTTCCCCAGTTCC	This Paper	N/A
TRAM2	CTTATTTGGTGCCATGCAATG	AGGGTGAGAAGGACCTTTAAGG	This Paper	N/A
GPCR6	CGTGTCCTCTCACCAACACC	AGCTCCACATCCTGAACACC	This Paper	N/A
ARG1	GTACTATGTGTCCATGTCATTC	TTAGTAGTTTTAAGCAGGACGTT	This Paper	N/A

Supplementary Table 2, related to Key Resource Table and STAR methods. Cloning and CRISPR primers.

Reagent or Resource	Source	Identifier
BTN2A-IgVgeno CRISPR Fwd (GCAGTGTTTGTGTATAAAGG)	This Paper	N/A
BTN2A-IgVgeno CRISPR Rev (CCTTTATACACAAACACTGC)	This Paper	N/A
BTN2A1geno 49FCRISPR Fwd (AATGAGGGGCCATGAAGACG)	This Paper	N/A
BTN2A1geno 49FCRISPR Rev (CGTCTTCATGGCCCCTCATT)	This Paper	N/A
BTN2A2geno 343CRISPR Fwd (TGAGGCCATCCTACGCCTCG)	This Paper	N/A
BTN2A2geno 343CRISPR Rev (CGAGGCGTAGGATGGCCTCA)	This Paper	N/A
pMIM-BTN2A1Fwd (CTTCTCTAGGCGCCGGAATTCGCCGCCACCATGGAATCAGCTGCTGCC)	This Paper	N/A
pMIM-BTN2A1Rev (TCCGCTAGCTACGTAAGATCTCTATAGGCTCTGGTGACC)	This Paper	N/A
pMIG-BTN2A2Fwd (AAATGAATTCGCCGCCACCATGGAACCAGCTGCTGCTC)	This Paper	N/A
pMIG-BTN2A2Rev (AAATAGATCTCTATAGGCTCTGGTGGGTCC)	This Paper	N/A
BTN2A1IgV Geno Fwd (CCCTTTGTTGAACAGCCCAG)	This Paper	N/A
BTN2A1IgV Geno Rev (CCAAAGTCACACTGTGCCAA)	This Paper	N/A
BTN2A2IgV Geno Fwd (CTGTAGCCTTGGAATATCTGCTTC)	This Paper	N/A
BTN2A2IgV Geno Rev (CTTTGTGCAAGAGGAAGCCACTTT)	This Paper	N/A
BTN2A1 49F Geno Fwd (ATGGGGCCTATAGGTGGACCG)	This Paper	N/A
BTN2A1 49F Geno Rev (AGGGTGTTGTTGATAGAGCAGGACATG)	This Paper	N/A
BTN2A2 343 Geno Fwd (AATATCTGCTTCCTTTCATCCCTG)	This Paper	N/A
BTN2A2 343 Geno Rev (GTGAAACCTATAGCTTAGCAGGGC)	This Paper	N/A
BTN2A1 R65A Fwd (AGGACATGGAGGTGGCGTGGTTCCGGTCTC)	This Paper	N/A
BTN2A1 R65A Rev (GAGACCGGAACCACGCCACCTCCATGTCCT)	This Paper	N/A
BTN2A1 R124A Fwd (AAAACGGCACCTACGCCTGTTACTTCCAAG)	This Paper	N/A
BTN2A1 R124A Rev (CTTGGAAGTAACAGGCGTAGGTGCCGTTTT)	This Paper	N/A
BTN2A1 Y126A Fwd (GCACCTACCGCTGTGCCTTCCAAGAAGGCA)	This Paper	N/A
BTN2A1 Y126A Rev (TGCCTTCTTGGAAGGCACAGCGGTAGGTGC)	This Paper	N/A
BTN2A1 R124E Fwd (CACCTACGAATGTTACTTCCAAGAAGGCAGG)	This Paper	N/A
BTN2A1 R124E Rev (GGAAGTAACATTCGTAGGTGCCGTTTTCC)	This Paper	N/A
BTN2A1 C247W Fwd (TGTGTCTCCCTGGGCAGTGGCCCTGCCTATC)	This Paper	N/A
BTN2A1 C247W Rev (GGGCCACTGCCCAGGGAGACACACTGGGC)	This Paper	N/A
pIZ-BTN2A1 Fwd (GACCATCCTCTAGAGAATTCGCCGCCACCATGGAATCAGCT)	This Paper	N/A
pIZ-BTN2A1-HA Rev (GGGAGGGAGAGGGGGATCCCTAAGCGTAATCTGGAACATCGTATGGGTATGCTGATCCTAGGCTCTGGTGGGTCCCCACT)	This Paper	N/A
pIZ-BTN2A2 Fwd (GACCATCCTCTAGAGAATTCGCCGCCACCATGGAACCAG)	This Paper	N/A
pIZ-BTN2A2-HA Rev (GGGAGGGAGAGGGGGATCCCTAAGCGTAATCTGGAACATCGTATGGGTATGCTGATCCTAGGCTCTGGTGGGTCCCCACTC)	This Paper	N/A
pIZ-BTN2A1Ldr-HA Fwd (ATGCCaaTTGGCCACCATGGAATCAGCTGCTGCCCTGCACTTCTCCCGGCCAGCCTCCCTCCTCCTCCTCCTCCTCAGCCTGTGTGCACTGGTCTCAGGATCAGCATACCCATACGA)	This Paper	N/A
pIZ-BTN2A1Ldr-HA Rev (TGTGTGCACTGGTCTCAGGATCAGCATACCCATACGATGTTCCAGATTACGCTGCTGGATCCACCATGGTAATTGAATTCAATATTTAAATAATAGATct)	This Paper	N/A
pIZ-HA-BTN2A1 Fwd (CCAGATTACGCTGCTGGATCCGCCCAGTTTATTGTCGTGG)	This Paper	N/A
pIZ-BTN2A1 Rev (TATTATTTAAATATTGAATTCCTATAGGCTCTGGTGGGTCC)	This Paper	N/A
pIZ-HA-BTN2A2 Fwd (CCAGATTACGCTGCTGGATCCGCCCAGTTTACTGTCGTGG)	This Paper	N/A
pIZ-BTN2A2-HA Rev (TATTATTTAAATATTGAATTCCTATAGGCTCTGGTGGGTCCC)	This Paper	N/A
pIH-BTN3A1Ldr-FLAG Fwd (GGATCAGCACAATTGGCCACCATGAAAATGGCAAGTTTCCTGGCCTTCCTTCTGCTCAACTTTCGTGTCTGCCTCCTTTTGCTTCAGCTGCTCATGCCTCACTCAGCTGGATCAGCAGACTACAA)	This Paper	N/A
pIH-BTN3A1Ldr-FLAG-Rev (TCCTGATGCAGATCTATTATTTAAATATTGAATTCAATTACCATGGTGGATCCAGCCTTGTCATCGTCGTCCTTGTAGTCTGCTGATCC)	This Paper	N/A
pIH-FLAG-BTN3A1 Fwd (CAAGGCTAGATCTCAGTTTTCTGTGCTTGGAC)	This Paper	N/A
pIH-BTN3A1 Rev (TATTGAATTCTCACGCTGGACAAATAGTCAG)	This Paper	N/A
MOP Vγ9 Fwd (GGACCATCCTCTAGAGAATTCGCCGCCACCATGCTGTCACTGCTGCTCC)	This Paper	N/A
MOP Vγ9 Rev (GGGGGAGGGAGAGGGGGATCCTTATGATTTCTCTCCATTGCAGCAG)	This Paper	N/A
MOP Vγ9-E70A Fwd (CAAATTTGCGGTGGATAGGATACCTGAAACGTC)	This Paper	N/A
MOP Vγ9-E70A Rev (TCCTATCCACCGCAAATTTGCCTGACGGAATG)	This Paper	N/A
MOP Vγ9-E70R Fwd (CAAATTTAGGGTGGATAGGATACCTGAAACGTC)	This Paper	N/A
MOP Vγ9-E70R Rev (TCCTATCCACCCTAAATTTGCCTGACGGAATG)	This Paper	N/A
MOP Vγ9-E70K Fwd (CAAATTTAAGGTGGATAGGATACCTGAAACGTC)	This Paper	N/A
MOP Vγ9-E70K Rev (TCCTATCCACCTTAAATTTGCCTGACGGAATG)	This Paper	N/A
