# Supplementary material for: Butyrophilin-2A1 Directly Binds Germline-Encoded Regions of the Vγ9Vδ2 TCR and Is Essential for Phosphoantigen Sensing
Source: Immunity. 2020 Mar 17;52(3):487–498.e6. doi: 10.1016/j.immuni.2020.02.014 (PMC7083227; doi:10.1016/j.immuni.2020.02.014)
Supplement: Document S2. Article plus Supplemental Information [file mmc2.pdf]

Immunity

Article

Butyrophilin-2A1 Directly Binds Germline-Encoded Regions of the Vγ9Vδ2 TCR and Is Essential for Phosphoantigen Sensing

Graphical Abstract

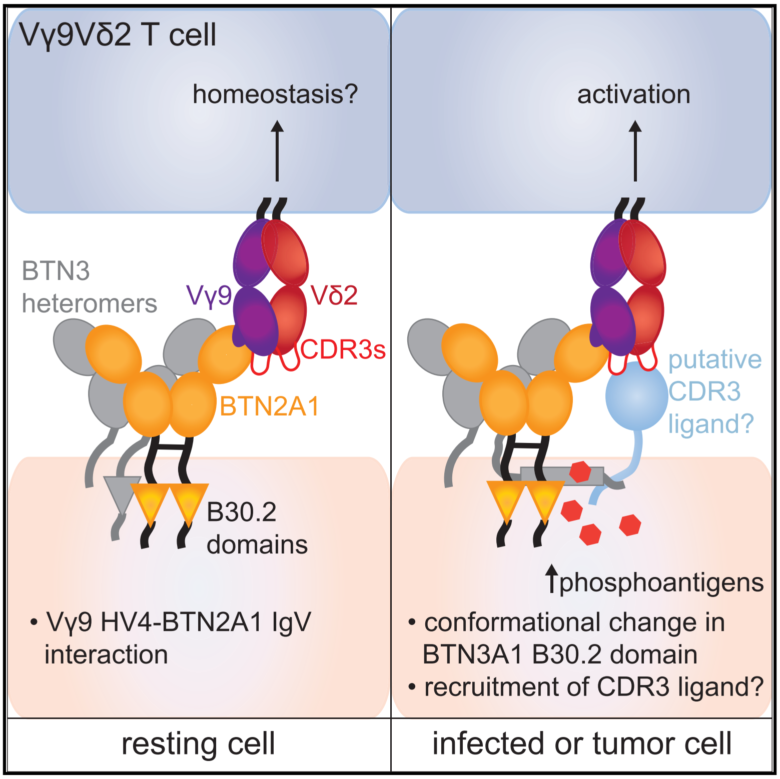

Highlights

- Radiation hybrids identify BTN2A1 as crucial for Vγ9Vδ2 phosphoantigen (P-Ag) sensing
- BTN2A1 binds directly to the T cell receptor via germline-encoded regions of Vγ9
- Cell-surface BTN2A1 associates directly with BTN3A1 independent of P-Ag stimulation
- The Vγ9-BTN2A1 interaction modality suggests an additional CDR3-dependent TCR ligand

Authors

Mohindar M. Karunakaran, Carrie R. Willcox, Mahboob Salim, ..., Fiyaz Mohammed, Benjamin E. Willcox, Thomas Herrmann

Correspondence

b.willcox@bham.ac.uk (B.E.W.), herrmann-t@vim.uni-wuerzburg.de (T.H.)

In Brief

Karunakaran et al. find that butyrophilin 2A1 (BTN2A1) associates with BTN3A1 on the cell surface and binds directly to germline-encoded regions of the Vγ9 chain of the Vγ9Vδ2 TCR. Thus, BTN2A1 collaborates with BTN3A1 to potentiate Vγ9Vδ2 T cell recognition, playing an essential role in phosphoantigen sensing.

Karunakaran et al., 2020, Immunity *52*, 487–498
March 17, 2020 © 2020 The Authors. Published by Elsevier Inc.
https://doi.org/10.1016/j.immuni.2020.02.014

CellPress

Butyrophilin-2A1 Directly Binds Germline-Encoded Regions of the Vγ9Vδ2 TCR and Is Essential for Phosphoantigen Sensing

Mohindar M. Karunakaran,[1,11] Carrie R. Willcox,[2,3,11] Mahboob Salim,[2,3] Daniel Paletta,[1] Alina S. Fichtner,[1] Angela Noll,[4] Lisa Starick,[1] Anna Nöhren,[1] Charlotte R. Begley,[2,3] Katie A. Berwick,[2,3] Raphaël A.G. Chaleil,[5] Vincent Pitard,[6,7] Julie Déchanet-Merville,[6,7] Paul A. Bates,[5] Brigitte Kimmel,[8] Timothy J. Knowles,[9] Volker Kunzmann,[8] Lutz Walter,[4] Mark Jeeves,[10] Fiyaz Mohammed,[2,3] Benjamin E. Willcox,[2,3,11,12,*] and Thomas Herrmann[1,11,*]
[1]Institute for Virology and Immunobiology, University of Würzburg, Würzburg, Germany
[2]Institute of Immunology and Immunotherapy, University of Birmingham, Birmingham, UK
[3]Cancer Immunology and Immunotherapy Centre, University of Birmingham, Birmingham, UK
[4]Primate Genetics Laboratory, German Primate Center, Leibniz Institute for Primate Research, Göttingen, Germany
[5]Biomolecular Modelling Laboratory, The Francis Crick Institute, London, UK
[6]ImmunoConcEpT Laboratory, Equipe labellisée, LIGUE 2017, UMR 5164, Bordeaux University, CNRS, 33076 Bordeaux, France
[7]Flow Cytometry Facility, TransBioMed Core, Bordeaux University, CNRS UMS 3427, INSERM US05, 33076 Bordeaux, France
[8]Medical Clinic and Policlinic II, University of Würzburg, Würzburg, Germany
[9]School of Biosciences, University of Birmingham, Birmingham, UK
[10]Henry Wellcome Building for NMR, Institute of Cancer and Genomic Sciences, University of Birmingham, Birmingham, UK
[11]These authors contributed equally
[12]Lead Contact
*Correspondence: b.willcox@bham.ac.uk (B.E.W.), herrmann-t@vim.uni-wuerzburg.de (T.H.)
https://doi.org/10.1016/j.immuni.2020.02.014

SUMMARY

Vγ9Vδ2 T cells respond in a TCR-dependent fashion to both microbial and host-derived pyrophosphate compounds (phosphoantigens, or P-Ag). Butyrophilin-3A1 (BTN3A1), a protein structurally related to the B7 family of costimulatory molecules, is necessary but insufficient for this process. We performed radiation hybrid screens to uncover direct TCR ligands and cofactors that potentiate BTN3A1's P-Ag sensing function. These experiments identified butyrophilin-2A1 (BTN2A1) as essential to Vγ9Vδ2 T cell recognition. BTN2A1 synergised with BTN3A1 in sensitizing P-Ag-exposed cells for Vγ9Vδ2 TCR-mediated responses. Surface plasmon resonance experiments established Vγ9Vδ2 TCRs used germline-encoded Vγ9 regions to directly bind the BTN2A1 CFG-IgV domain surface. Notably, somatically recombined CDR3 loops implicated in P-Ag recognition were uninvolved. Immunoprecipitations demonstrated close cell-surface BTN2A1-BTN3A1 association independent of P-Ag stimulation. Thus, BTN2A1 is a BTN3A1-linked co-factor critical to Vγ9Vδ2 TCR recognition. Furthermore, these results suggest a composite-ligand model of P-Ag sensing wherein the Vγ9Vδ2 TCR directly interacts with both BTN2A1 and an additional ligand recognized in a CDR3-dependent manner.

INTRODUCTION

Human peripheral blood γδ T cells are dominated from an early age by Vγ9Vδ2 lymphocytes (Parker et al., 1990), an innate-like subset that features a predominant effector status, allowing potent cytokine production and cytotoxic capability that is linked to a relatively restricted T cell receptor (TCR) repertoire (Davodeau et al., 1993; Delfau et al., 1992). Vγ9Vδ2 T cells universally respond in a TCR-dependent fashion to non-peptidic pyrophosphate compounds (phosphoantigens [P-Ag]). These include the microbially derived compound (E)-4-hydroxy-3-methyl-but-2-enyl pyrophosphate (HMBPP) (Morita et al., 2007), which is generated by the non-mevalonate isoprenoid synthetic pathway and is a highly potent activator of Vγ9Vδ2 T cells. In addition, host-cell-derived isoprenyl pyrophosphate (IPP) can act as a P-Ag and stimulate Vγ9Vδ2 T cell responses. IPP levels are elevated in some cancer cells and can also be therapeutically increased in target cells via aminobisphosphonate drugs that inhibit IPP catabolism, such as Zoledronate (Zol) (Gober et al., 2003; Kunzmann et al., 2000).

Vγ9Vδ2-mediated P-Ag sensing requires cell-cell contact (Morita et al., 1995) and depends on both Vγ and Vδ chains, with evidence for involvement of multiple complementarity-determining region (CDR) loops (Wang et al., 2010). An essential prerequisite for P-Ag sensing is target-cell expression of butyrophilin (BTN) 3A1 (Harly et al., 2012), a member of a multi-gene family encoded on chromosome (Chr) 6. BTNs and butyrophilin-like (BTNL) molecules are structurally related to the B7 family of costimulatory molecules, comprising two extracellular immunoglobulin (Ig)-like domains, a transmembrane region, and a cytoplasmic tail that often contains a B30.2 domain (Rhodes

This is an open access article under the CC BY license (http://creativecommons.org/licenses/by/4.0/).

et al., 2016). In addition to immunomodulatory effects on antigen-presenting cells and conventional αβ T cells, several BTN and/or BTNL family members are emerging as playing critical roles in γδ T cell development and activation (Boyden et al., 2008; Di Marco Barros et al., 2016; Harly et al., 2012; Melandri et al., 2018; Vantourout et al., 2018; Willcox et al., 2019). Although the extracellular domain of BTN3A1 was initially reported to present P-Ag and directly bind the Vγ9Vδ2 TCR (Vavassori et al., 2013), other studies have challenged both of these findings and instead support the concept that BTN3A1 senses P-Ag directly. These data include robust evidence for P-Ag binding to the intracellular B30.2 domain of BTN3A1 and for a P-Ag-induced conformational change (Nguyen et al., 2017; Salim et al., 2017; Sandstrom et al., 2014). In addition, the importance of BTN3A2 and/or BTN3A3 co-expression alongside BTN3A1 for optimal P-Ag sensing has been highlighted, as well as the potential of these family members to heterodimerize with BTN3A1 in an IgC-dependent manner (Vantourout et al., 2018).

Vγ9Vδ2 T cells emerged with the appearance of placental mammals and have been retained in both primates and species as diverse as dolphin (*Tursiops truncatus*) and alpaca (*Vicugna pacos*) (Fichtner et al., 2018; Karunakaran et al., 2014). Of note, the alpaca is the only non-primate species to date with proven P-Ag reactivity of Vγ9Vδ2 T cells and P-Ag binding to BTN3 demonstrated (Fichtner et al., 2020). In contrast, rodents lack BTN3, Vδ2, and Vγ9 homologs (Karunakaran et al., 2014). Consistent with an important role in host immunity, Vγ9Vδ2 T cell expansion and activation is observed in a variety of microbial infections (Morita et al., 2007). Furthermore, attempts to therapeutically harness the human γδ T cell compartment have hitherto focused predominantly on the Vγ9Vδ2 subset in the context of both specific infections (Shen et al., 2019) and cancer (Kunzmann et al., 2000; Silva-Santos et al., 2019). From this perspective, the mechanism underpinning Vγ9Vδ2 T cell activation has been a focus of strong interest.

Our previous studies have established that BTN3A1 is necessary but not sufficient for P-Ag sensing and indicated the existence of an additional putative Chr-6-encoded factor that synergized with BTN3A1 to stimulate P-Ag-mediated responses (Riaño et al., 2014), which we subsequently coined "Factor X" (Karunakaran and Herrmann, 2014). Here, we set out to identify Factor X using a radiation hybrid approach. We identified BTN2A1 as this critical factor and showed it interacts directly with the Vγ9Vδ2 TCR to potentiate P-Ag-dependent recognition, highlighting its role in a "composite ligand" model of Vγ9Vδ2 T cell recognition.

RESULTS

Radiation Hybrids Identify BTN2A1 as Essential for P-Ag Sensing

We showed previously that a T cell hybridoma expressing the Vγ9Vδ2 MOP TCR produced interleukin (IL)-2 in co-culture with BTN3A1-transduced Chinese hamster ovary (CHO) cells incubated with the anti-BTN3A1 monoclonal antibody (mAb) 20.1 but exhibited a complete lack of response to HMBPP or Zol (Riaño et al., 2014; Starick et al., 2017). In contrast, HMBPP and Zol sensitivity was restored in co-cultures with human-rodent hybrid cells, including CHO cells containing a single human Chr 6 (CHO Chr6 cells). Based on this observation, we postulated the existence of a Factor X encoded on Chr 6, which in addition to BTN3A1 is mandatory for P-Ag-mediated γδ T cell stimulation (Riaño et al., 2014).

To identify Factor X, we used an unbiased genome-based approach involving generation of radiation hybrids between CHO-Chr 6 cells and BTN3A1-transduced hypoxanthine-aminopterin-thymidine (HAT)-sensitive rodent fusion partners and subsequent analysis of their capacity to stimulate P-Ag sensing by Vγ9Vδ2 T cells (Figure 1A). We postulated that comparison of the human gene products transcribed in stimulatory radiation hybrids would allow mapping of the gene(s), which alongside BTN3A1 are mandatory for PAg-mediated stimulation.

We fused CHO-Chr6 cells separately with two HAT-sensitive fusion partners—first BTN3A1-transduced A23 hamster cells and second BTN3A1-transduced mouse BW cells (STAR Methods) (Sanderson and Shastri, 1994); resulting radiation hybrids were assessed for P-Ag-dependent activation of TCR-MOP transductants and positive candidates cloned by limiting dilution. In some cases, these clones were used as donor cells for further fusions. A final selection of clones (Figure S1A) were subjected to RNA sequencing (RNA-seq) analysis (Figure 1B; STAR Methods) alongside CHO-Chr6 cells and rodent fusion partner cells as positive and negative controls, respectively.

A region of ~580 kB of Chr 6 permitting P-Ag-mediated stimulation by the radiation hybrids was identified (Figures 1C and S1B). Analysis of candidate genes within this region revealed that the only transmembrane molecules among the expressed human genes were the major histocompatibility complex (MHC)-class-I-like iron transporter *HFE*, the *BTN3A1* gene already transduced into rodent fusion partners, *BTN3A2*, *BTN3A3*, and *BTN2A1* and *BTN2A2*. Since we knew that expression of all three BTN3 genes was insufficient for reconstitution of the P-Ag response (D.P., A.S.F., M.M.K., and T.H., unpublished data), *BTN2A1* and *BTN2A2*, which to date have been discussed mainly for their immunomodulatory properties (Rhodes et al., 2016), emerged as the prime candidates for encoding Factor X.

We then tested the effects on P-Ag-dependent stimulation of Vγ9Vδ2 lymphocytes by human 293T cells after CRISPR-Cas9-mediated inactivation of either both BTN2 genes ($BTN2^{-/-}$) or *BTN2A1* ($BTN2A1^{-/-}$) or *BTN2A2* alone ($BTN2A2^{-/-}$). Inactivation of both BTN2 genes completely abolished interferon (IFN)γ production by polyclonal Vγ9Vδ2 T cell lines in response to Zol pulsed cells (Figure 1D). Crucially, both $BTN2^{-/-}$ and $BTN2A1^{-/-}$ exhibited a complete loss of IL-2 production by TCR-MOP cells in response to either HMBPP (Figure 1E) or 20.1 mAb (Figure 1F), whereas responses to $BTN2A2^{-/-}$ were similar to wild-type (WT) 293T cells (Figures 1E and 1F). These experiments strongly suggested that alongside BTN3A1, BTN2A1 was critical for P-Ag sensing.

BTN2A1 and BTN3A1 Are Sufficient to Potentiate Vγ9Vδ2-Mediated P-Ag Sensing

To address whether *BTN2A1* was sufficient alongside *BTN3A1* to reconstitute P-Ag sensitization in rodent cells, we transduced either one or both genes into both $CD80^+$ BW and $CD80^+$ CHO cells (Figures 2A–2C) and tested their ability to induce IL-2 production from TCR-MOP cells following incubation with HMBPP. In both cases, whereas transduction of BTN3A1 alone resulted in

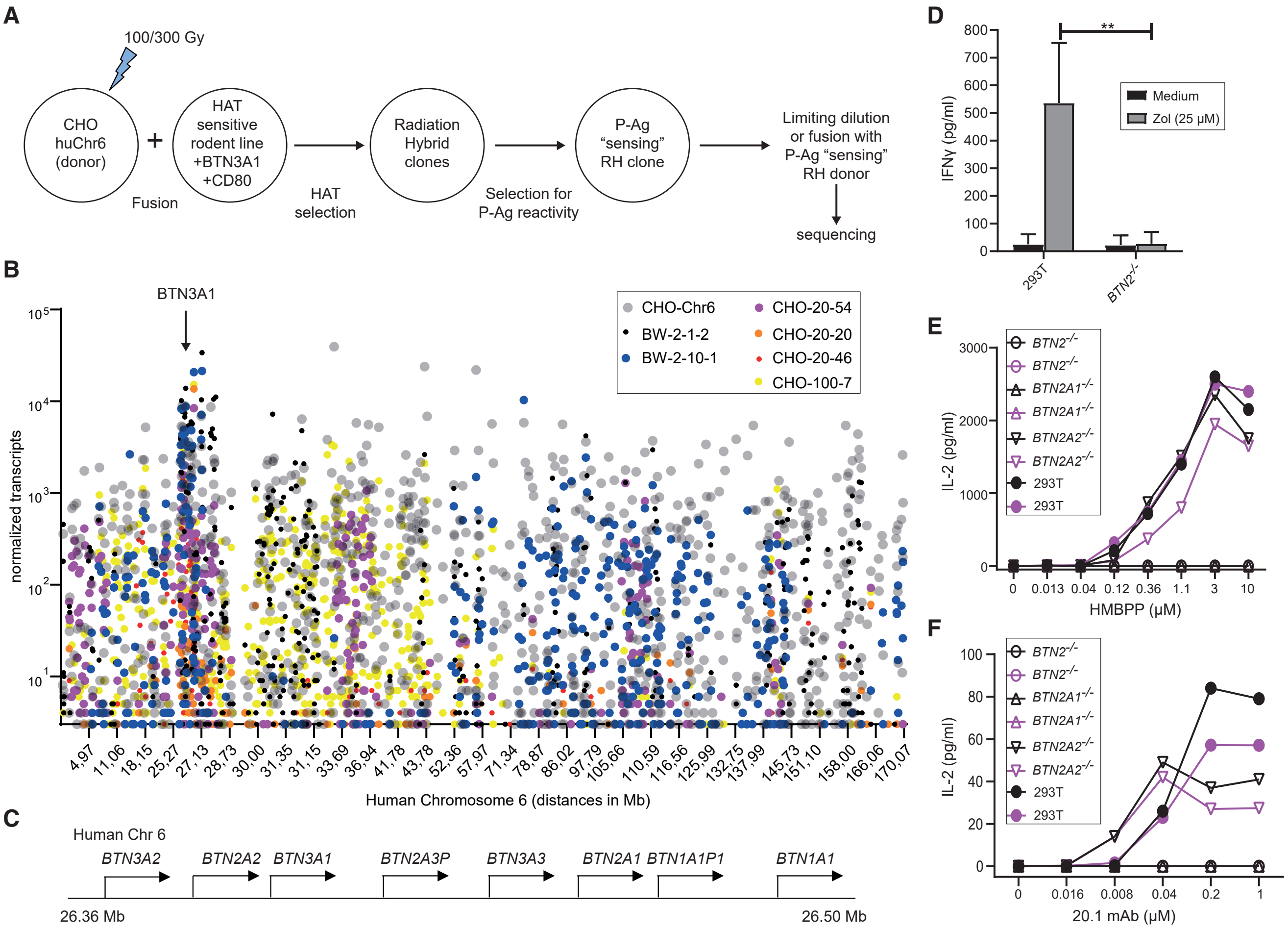

Figure 1. Identification of BTN2A1 as Factor X

(A) Radiation hybrid approach to generate and identify rodent cell-fusion hybrids incorporating portions of human chromosome (Chr) 6 that permit P-Ag sensitization.

(B) RNA-seq analysis of prioritized clones generated from fusion with A23 or BW cells. Values for less than three transcripts are merged with the x axis.

(C) Arrangement of BTN gene cluster on Chr 6 extracted from genome data viewer GRCh38.p13 (GCF_000001405.39).

(D) Production of IFNγ from polyclonal Vγ9Vδ2 T cell lines in response to Zol-treated WT or *BTN2*$^{-/-}$ 293T cells. Error bars represent standard deviation for three independent experiments. **p < 0.005.

(E) Production of IL-2 from TCR-MOP transductants in response to HMBPP-treated WT, *BTN2*$^{-/-}$, *BTN2A1*$^{-/-}$, and *BTN2A2*$^{-/-}$ 293T cells.

(F) Production of IL-2 from TCR-MOP transductants in response to 20.1 mAb-treated WT, *BTN2*$^{-/-}$, *BTN2A1*$^{-/-}$, and *BTN2A2*$^{-/-}$ 293T cells.

In (E) and (F), the different colors indicate results from two independent experiments. See also Figure S1.

negligible responses, transduction of both BTN2A1 and BTN3A1 permitted a robust, HMBPP-dose-dependent IL-2 response, confirming their sufficiency for P-Ag sensitization (Figures 2B and 2C). Interestingly, transduction of BTN2A1 alone resulted in a weak, HMBPP-dose-independent basal response to both cell lines (Figures 2B and 2C).

To assess whether BTN2A1 surface expression was able to support binding to the Vγ9Vδ2 TCR, we generated Vγ9Vδ2 TCR tetramers and used them to stain transduced BW and 293T cells (Figures 2D and 2E). BTN2A1 expression on transduced BW and 293T cells was sufficient to enable staining by Vγ9Vδ2 MOP-TCR tetramer (Figures 2D, 2E, and S2A), supporting the idea that BTN2A1 may be a direct TCR ligand; moreover, all Vγ9Vδ2 TCR tetramers tested stained BTN2A1-transduced cells (Figure 2E). Consistent with the minimal basal BTN2A1-dependent IL-2 response observed in the absence of P-Ag or BTN3A1 (Figures 2A and 2B), BTN3A1 co-expression was not required for BTN2A1-mediated tetramer staining (Figure 2D), nor was exposure to Zol necessary for tetramer staining (Figure 2F). This suggested BTN2A1 might be an independent ligand for the Vγ9Vδ2 TCR, the activatory potential of which is critically augmented in a BTN3A1- and P-Ag-dependent manner.

We then investigated why BTN2A2, which shares close 88% sequence identity with BTN2A1 in its extracellular region, was unable to potentiate P-Ag sensing alongside BTN3A1. BTN2A2-293T transductants did not support tetramer staining (Figure S2A), suggesting BTN2A2 might not be able to recognize the Vγ9Vδ2 TCR. However, one major caveat was the considerably lower surface expression of BTN2A2 relative to BTN2A1 in 293T transductants (Figure S2B), which could also explain this observation.

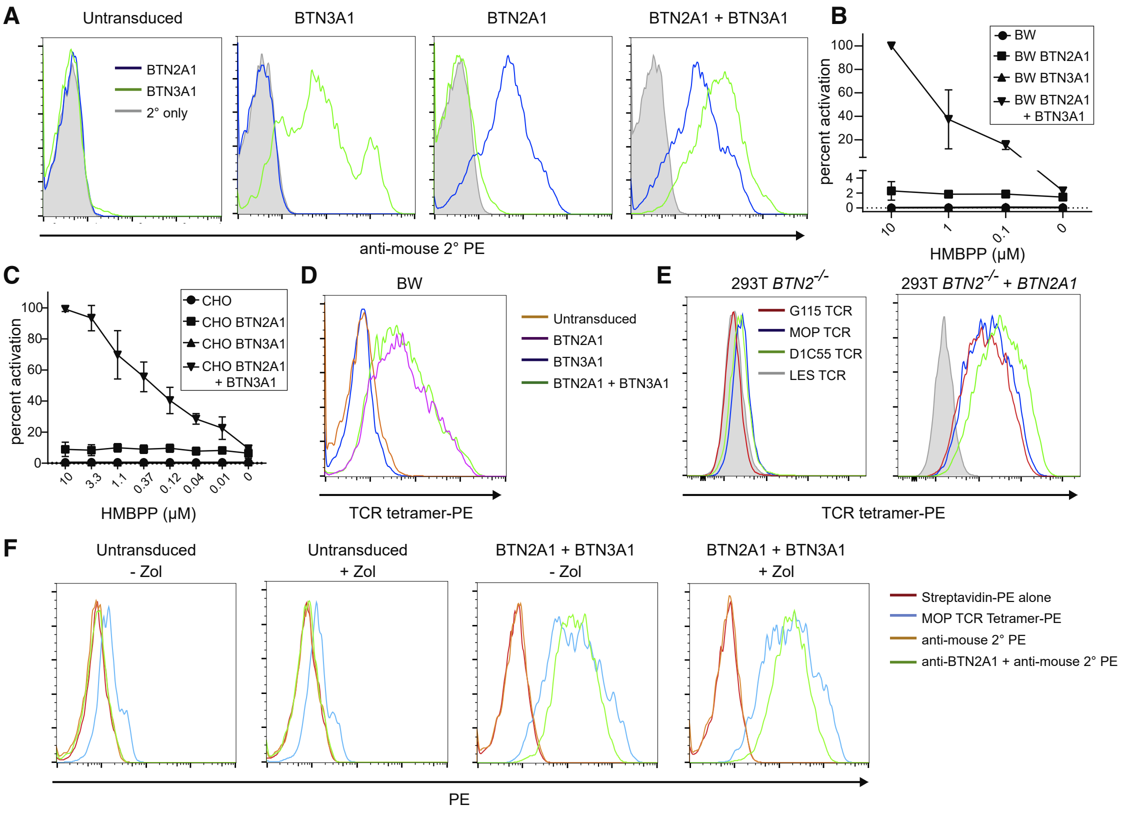

Figure 2. BTN2A1 and BTN3A1 Synergize to Potentiate P-Ag Sensing in Rodent Cells

(A) Expression of BTN2A1, BTN3A1, or both genes in transduced BW cells.

(B) Production of IL-2 by TCR-MOP transductants in response to HMBPP-treated $CD80^+$ BW cells transduced to express BTN2A1, BTN3A1, both, or untransduced controls. Percentage activation is normalized against the maximum response obtained from $CD80^+$ CHO cells expressing both BTN2A1 and BTN3A1 in the presence of 10 μM HMBPP.

(C) Production of IL-2 from TCR-MOP transductants in response to HMBPP-treated $CD80^+$ CHO cells transduced with either BTN2A1, BTN3A1, both genes, or untransduced controls, with responses normalized as in (B).

Error bars in (B) and (C) represent standard deviation for three independent experiments. Differences between untransduced and BTN2-transduced cells were significant ($p < 0.05$), as were those between the BTN2A1-transductant and BTN2A1+BTN3A1-transductant in the presence of HMBPP.

(D) MOP-TCR tetramer staining of transduced BW cells.

(E) Staining of transduced 293T cells with $V\gamma9V\delta2$ TCRs.

(F) MOP-TCR tetramer staining or anti-BTN2A1 mAb staining of BTN2A1 and BTN3A1-transduced $CD80^+$ BW cells versus untransduced controls in the presence and absence of Zol.

See also Figure S2.

BTN2A1 IgV Domain Directly Binds Germline-Encoded Regions of $V\gamma9^+$ TCRs

To establish whether BTN2A1 acted as a direct ligand for the $V\gamma9V\delta2$ TCR, we expressed the membrane-distal domain of the BTN2A1 ectodomain and tested direct binding to recombinant $V\gamma9V\delta2$ TCR using surface plasmon resonance (SPR). Injection of BTN2A1 IgV produced substantially enhanced signals over surfaces with immobilized $V\gamma9V\delta2$ TCR relative to $V\gamma4V\delta5$ and $V\gamma2V\delta1$ TCRs or control streptavidin surfaces, indicating specific binding (Figure 3A). Equilibrium affinity measurements of BTN2A1 IgV binding to the G115 and MOP $V\gamma9V\delta2$ TCRs established K_d values of 45.4 μM (n = 9) and 49.9 μM (n = 8), respectively (Figure 3B).

Unexpectedly, experiments also indicated clear binding of BTN2A1 IgV to a $V\gamma9V\delta1$ TCR, which was derived from the non-P-Ag reactive $V\delta1^+$ T cell subset (Halary et al., 2005) (Figure 3A); consistent with this, $V\gamma9V\delta1$ TCR tetramers specifically stained BTN2A1-transduced 293T cells (Figure S3A). Taking into account the highly similar affinity (K_d 46.6μM [n = 8]) of the BTN2A1-$V\gamma9V\delta1$ TCR interaction (Figure 3B) and the radically divergent CDR3γ expressed by this TCR relative to $V\gamma9V\delta2$ TCRs (Figure S3A), these results strongly suggested the BTN2A1-$V\gamma9V\delta2$ interaction focused on germline encoded regions of the Vγ9 IgV domain. This implied that the BTN2A1-$V\gamma9V\delta2$ interaction might be analogous to BTNL3 binding to human $V\gamma4^+$ TCRs (Melandri et al., 2018; Willcox et al., 2019), which is similarly focused on germline-encoded regions of the Vγ4 chain and allowed us to model BTN2A1-Vγ9 interaction based on the proposed BTNL3-Vγ4 interaction mode (Figure 3C).

An initial homology model suggested strong feasibility of a similar interaction mode and highlighted seven amino acids on the face of the BTN2A1 IgV domain incorporating the C, C', F

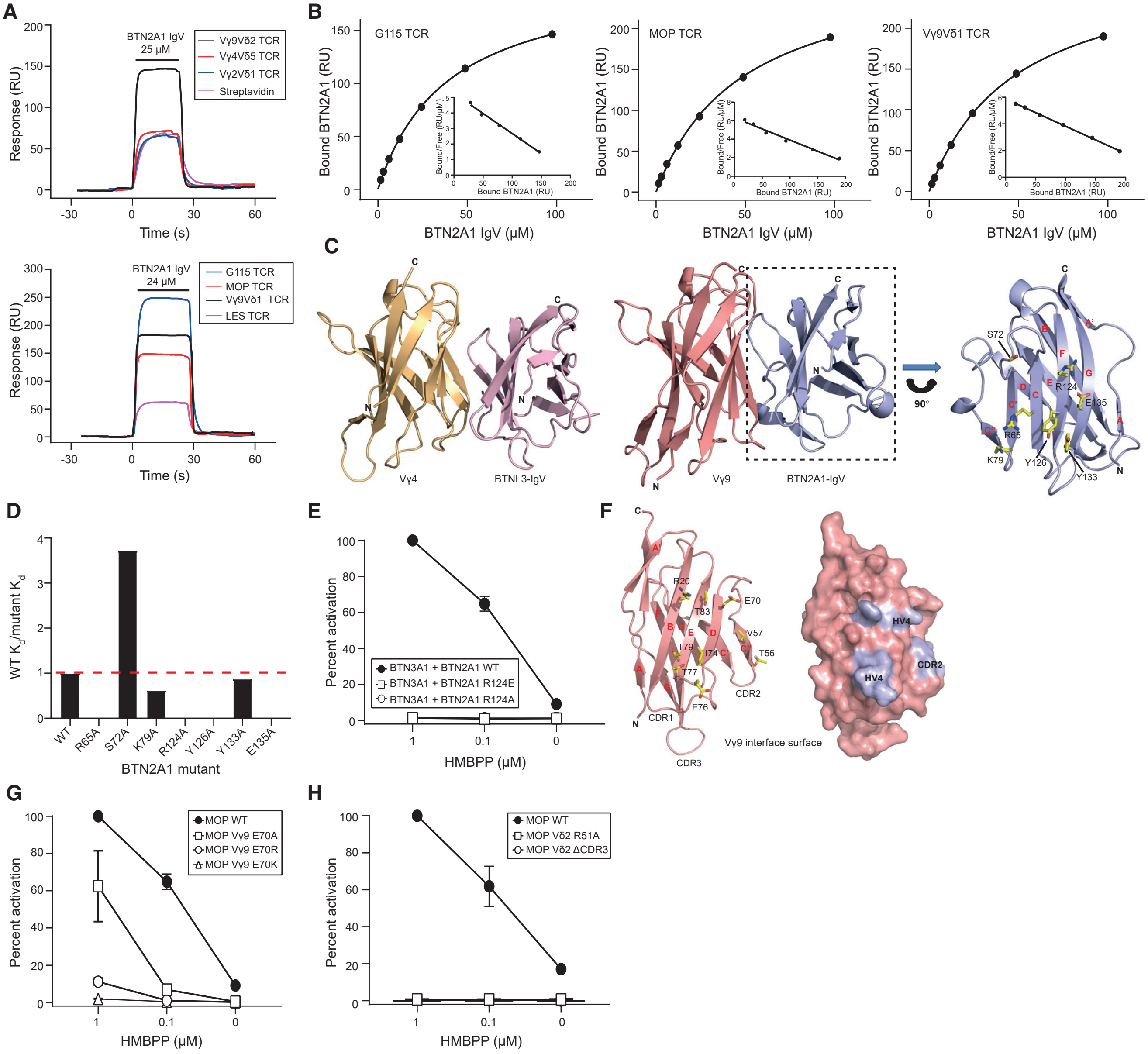

Figure 3. Direct BTN2A1 Binding to Germline-Encoded Regions of Vγ9 Is Essential for P-Ag Sensing

(A) (Top panel) Injection of BTN2A1 IgV (25 μM) over surfaces with immobilized Vγ9Vδ2 TCR (2,457 resonance units (RU)) and control surfaces comprising Vγ4Vδ5 TCR (2,351 RU), Vγ2Vδ1 TCR (1,800 RU), or streptavidin alone. Notably, signals over streptavidin alone and control TCR surfaces are equivalent. (Bottom panel) Injection of BTN2A1 IgV (24 μM) over surfaces with immobilized G115 (Vγ9Vδ2; 3,109 RU), MOP (Vγ9Vδ2; 3,108 RU), and Vγ9Vδ1 (2,774 RU) TCRs and LES TCR control (Vγ4Vδ5; 2,885 RU).

(B) Equilibrium affinity measurements and Scatchard analysis (inset) of BTN2A1 IgV binding to the G115 (K_d = 39.5 μM) and MOP (K_d = 48.4 μM) Vγ9Vδ2 TCRs and Vγ9Vδ1TCR (K_d = 47.9 μM).

Data in (A) and (B) are representative of eight to nine independent experiments.

(C) Model of the BTN2A1-Vγ9 interaction mode based on the proposed BTNL3-Vγ4 interaction, with expanded panel showing potential contacts at the Vγ9-BTN2A1 IgV interface.

(D) Effects of seven alanine substitutions in proposed BTN2A1 interface residues on Vγ9Vδ2 TCR interaction, indicating affinity of mutant BTN2A1 relative to WT BTN2A1 calculated in the same experiment. Data shown are representative of two independent experiments.

(E) Effects of BTN2A1 R124A and R124E mutations on IL-2 production by TCR-MOP in response to HMBPP-treated BTN3A1 and BTN2A1 expressing $CD80^+$ CHO cells.

(F) Predicted involvement of Vγ9 HV4 and CDR2 residues in BTN2A1 interaction.

(G) Effects of Vγ9-E70 mutation (HV4) on IL-2 production by TCR-MOP in response to HMBPP-treated BTN3A1 and BTN2A1 expressing $CD80^+$ CHO cells.

(legend continued on next page)

and G β strands (CFG face), equivalent to the region of BTNL3 IgV domain involved in binding Vγ4, as candidates for alanine mutation (Figure 3C). Individual BTN2A1 alanine mutants were generated for these seven residues. Of these, four completely abrogated BTN2A1 binding to Vγ9Vδ2 TCR (R65A, R124A, Y126A, E135A), a fifth marginally decreased affinity (K79A), Y133A did not affect binding, and S72A increased affinity (K_d 10–15 μM) (Figures 3D and S3B). These results allowed generation of an improved, mutationally informed model of BTN2A1/Vγ9 interaction using the high-ambiguity driven protein-protein DOCKing (HADDOCK) software, analysis of which outlined a molecular rationale for the effect of each mutation (Figure S3C; STAR Methods). Based on comparison of the BTN2A2 IgV sequence (Figure S3D) and a BTN2A2 homology model (Figure S3E) in the context of this BTN2A1 model, we predicted that BTN2A2 IgV would also be competent for Vγ9 TCR binding, which was subsequently confirmed using SPR for both Vγ9Vδ2 TCRs (Figures S3F and S3G) and a Vγ9Vδ1 TCR (Figures S3G and S3H), which indicated a similar affinity to BTN2A1 (K_d 39–50 μM [n = 3]).

To assess the dependence of the functional activity of BTN2A1 on TCR binding, we transduced CHO-BTN3A1 cells with the BTN2A1 R124A mutation shown to abrogate Vγ9$^+$ TCR binding (and also a BTN2A1 R124E charge-reversal mutant) and assessed effects on Vγ9Vδ2-mediated P-Ag response. Although permissive for cell-surface BTN2A1 expression (Figure S3I), both mutations completely abrogated both P-Ag-dependent IL-2 production and basal P-Ag-independent BTN2A1-mediated responses (Figure 3E). Furthermore, BTN2A1 R65A and Y126A mutations that eliminated Vγ9$^+$ TCR-BTN2A1 interaction also abrogated P-Ag-dependent and independent responses (Figure S3J). However, although mCherry reporter signal was detected for each construct, it must be noted that these mutant proteins could not be detected using the anti-BTN2A1 mAb (Figures S3K and S3L). We therefore could not exclude the possibility that these mutations affected cell-surface expression, although alternatively, they could be important components of the anti-BTN2A1 mAb epitope.

The mutationally guided model also indicated involvement of multiple TCR residues in the HV4 (including E70, I74, E76, T77, T79) and CDR2 (G56, T57, V58) loops of the Vγ9 IgV domain in BTN2A1 interaction, regions also critical for BTNL3-Vγ4 interaction (Willcox et al., 2019) (Figure 3F). Consistent with this, Vγ9Vδ2-expressing hybridomas bearing mutations at TCRγ HV4 E70 eliminated BTN2A1-dependent P-Ag-independent IL-2 production and substantially affected TCR-dependent P-Ag responses, with E70K exhibiting severely reduced activation potential (Figure 3G). Although the BTN2A1-Vγ9 model was supported by our BIAcore data (Figure 3A) in indicating no role for Vδ in BTN2A1 recognition, we sought to establish whether BTN2A1-dependent P-Ag sensing was nevertheless affected by Vδ2 CDR loops by generating TCR hybridomas bearing either a CDR3 deletion of TCR-MOP (ΔCDR3) (Figure S3B) or a R51A substitution in CDR2 (Li, 2010). Each mutation abolished both P-Ag-dependent IL-2 responses and BTN2A1-dependent P-Ag-independent basal responses (Figure 3H).

Collectively, these findings established that Vγ9Vδ2 TCR binds BTN2A1 IgV via a binding mode that closely mimics that of Vγ4 TCR for BTNL3 and that this binding is essential for P-Ag sensing but occurs alongside parallel and essential Vδ2 CDR-mediated binding events.

BTN2A1 Can Form Disulphide-like Homodimers at the Cell Surface

BTN and BTNL molecules have been shown to form either homo- or heterodimers (Palakodeti et al., 2012; Vantourout et al., 2018). To investigate BTN2A1's propensity for dimer formation, we carried out homology modeling of BTN2A1 IgV-C (Figures 4A and 4B) based on superposition of BTN2A1 onto the structure of the BTN3A1 V-shaped homodimer (Palakodeti et al., 2012). Inspection of the model confirmed a viable IgC-IgC homodimer interface driven by main-chain-main-chain hydrogen bonding interactions supplemented by side-chain-dependent hydrophobic contacts; these were predicted to be broadly equivalent to those of BTN3A1 IgC-IgC, albeit with increased interchain hydrophobic contacts in BTN2A1 (M153, F235) versus BTN3A1 (V154, S236), indicating a strong potential for non-covalent dimer formation (Figure S4A); in addition, further analyses indicated a similar potential for heterodimer formation with other members of the BTN family (Figure S4B).

Interestingly, the BTN2A1 IgV-C model also highlighted close proximity of extracellular membrane-proximal cysteine residue (C247) with its equivalent residue in the opposing monomer (Figure 4B), suggesting that the homodimer might be stabilized additionally by an interchain disulphide bond; of note, this cysteine is lacking in all other BTN and BTNL molecules (Figure S4C). Indeed, SDS-PAGE and immunoblot-streptavidin detection of BTN2A1 under reducing and non-reducing conditions confirmed that the overwhelming majority of cell surface BTN2A1 was present as a disulphide-bonded dimer (Figure 4C), even in the presence of BTN3A1 and in the presence and absence of Zol (Figure S4D), consistent with the BTN2A1 homodimer model (Figures 4A and 4B). However, transduction of BTN2A1 bearing a C247W mutation did not affect P-Ag-dependent or P-Ag-independent basal IL-2 production (Figure 4D); therefore, any disulphide stabilization may be redundant owing to strong existing non-covalent homodimer potential. In contrast, BTN2A2, which lacks this cysteine residue, did not form disulphide-linked dimers (Figure 4C), but analogous structural modeling indicated equivalent propensity for non-covalent IgC-IgC-mediated homodimer formation (Figure S4E).

Finally, by combining our HADDOCK-derived model of Vγ9Vδ2/BTN2A1 interaction (Figure S3B) with our BTN3A1-based homology model of the BTN2A1 homodimer (Figure 4A), we were able to envisage how BTN2A1 recognition might take place at the cell surface (Figure 4E). Notably, the Vγ9Vδ2 TCR-BTN2A1 interaction mode can in principle allow clustering of

(H) Effects of mutations in Vδ2 CDR2 (R51A) or a deletion in CDR3 (ΔCDR3) on IL-2 production by TCR-MOP in response to HMBPP-treated BTN3A1 and BTN2A1 expressing CD80$^+$ CHO cells.

In (E), (G) and (H), error bars indicate standard deviation for three independent stimulation experiments. Percentage activation is normalized against the maximum response obtained from CHO cells expressing both BTN2A1 and BTN3A1 in the presence of 1 μM HMBPP. Differences between WT and mutants in (E), (G), and (H) were significant, except for TCR-MOP E70A at 1 μM. See also Figure S3.

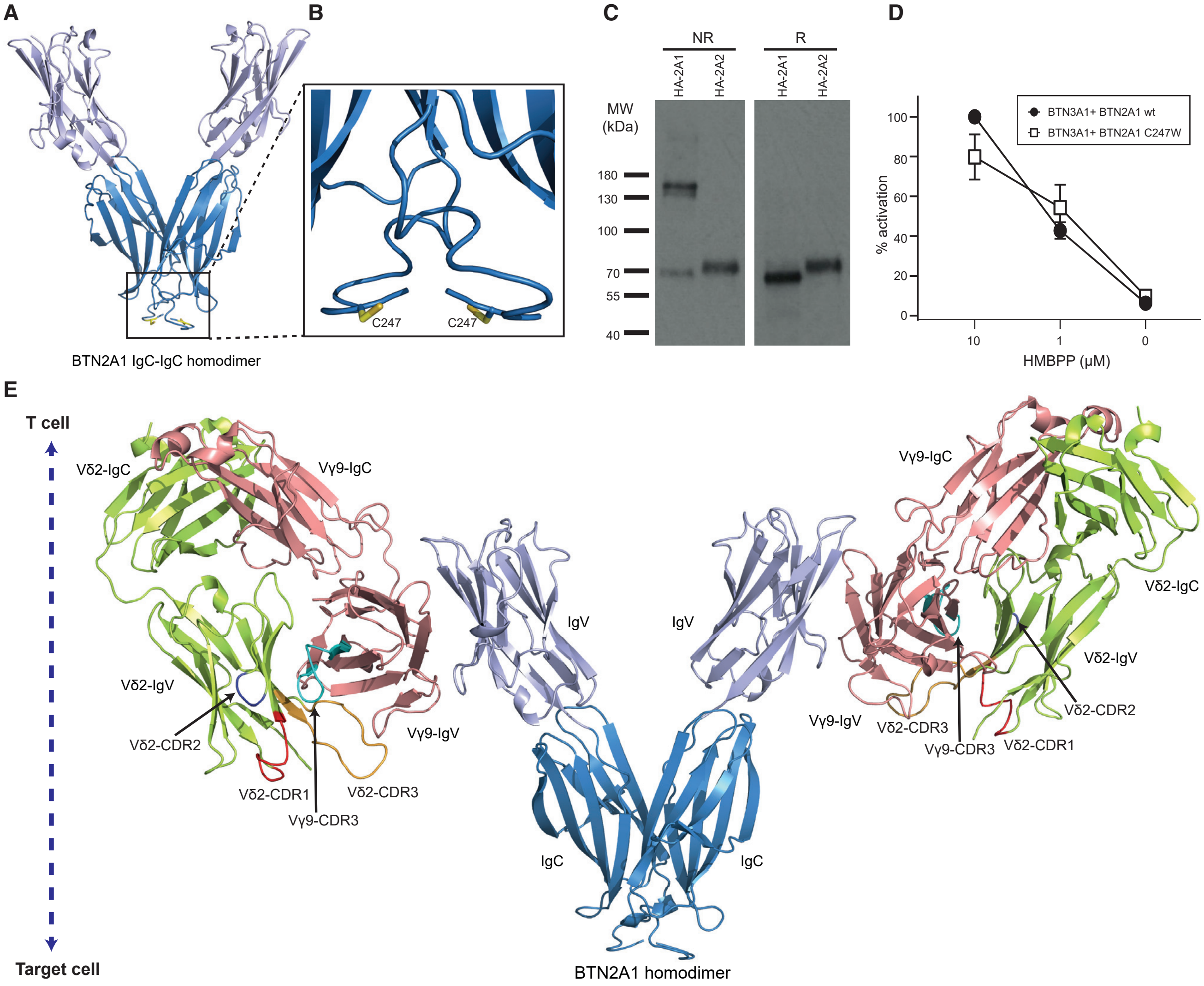

Figure 4. BTN2A1 Forms Disulphide-Linked Homodimers at the Cell Surface

(A) Homology model of BTN2A1 homodimer.

(B) C-terminal region of BTN2A1 homology model indicating close proximity of Cys residues.

(C) Non-reducing (NR) or reducing (R) SDS-PAGE analysis of CHO-cell expressed BTN2A1 and BTN2A2 protein.

(D) Effects of BTN2A1C247W mutation on IL-2 production by TCR-MOP in response to HMBPP-treated CD80$^+$ CHO cells expressing BTN2A1 and BTN3A1. Error bars indicate standard deviation for three independent stimulation experiments. Percentage activation is normalized against the maximum response obtained from CHO cells expressing both BTN2A1 and BTN3A1 in the presence of 1 μM HMBPP.

(E) Model of Vγ9Vδ2-BTN2A1 interaction incorporating BTN2A1 homodimer formation, and bilateral Vγ9Vδ2 interaction with BTN2A1 IgV domain.

See also Figure S4.

two TCRs for each BTN2A1 homodimer, each with somatically recombined CDR3 loops implicated in P-Ag sensing oriented directly toward the target cell surface.

BTN2A1 Is Closely Associated with BTN3A1 at the Cell Surface

To assess whether BTN2A1 and BTN3A1 were associated with each other at the cell surface either before or after P-Ag exposure, a membrane-impermeable amine-reactive cross-linker incorporating a 16Å spacer was used to cross-link proteins on the surface of CHO transductants co-expressing C-terminally HA-tagged BTN2A1 (BTN2A1-HA) and a N-terminally FLAG-tagged BTN3A1 (FLAG-BTN3A1). Immunoprecipitation (IP) using anti-HA beads or 20.1 mAb and subsequent anti-FLAG western blot (WB) was used to detect cross-linked BTN2A1-BTN3A1 species (Figure 5).

Following IP of BTN2A1-HA using anti-HA beads and subsequent WB detection of FLAG-BTN3A1 under reducing conditions using an anti-FLAG antibody, two discrete bands were detected that considerably exceeded the size of either BTN2A1 or BTN3A1 monomers (observed molecular weights [MWs] ~55k Da, FLAG-BTN3A1; ~70 kDa, BTN2A1-HA) (Figure 5A); each was only detected in the presence of cross-linker. One band was ~130 kDa, equivalent to cross-linking of a single

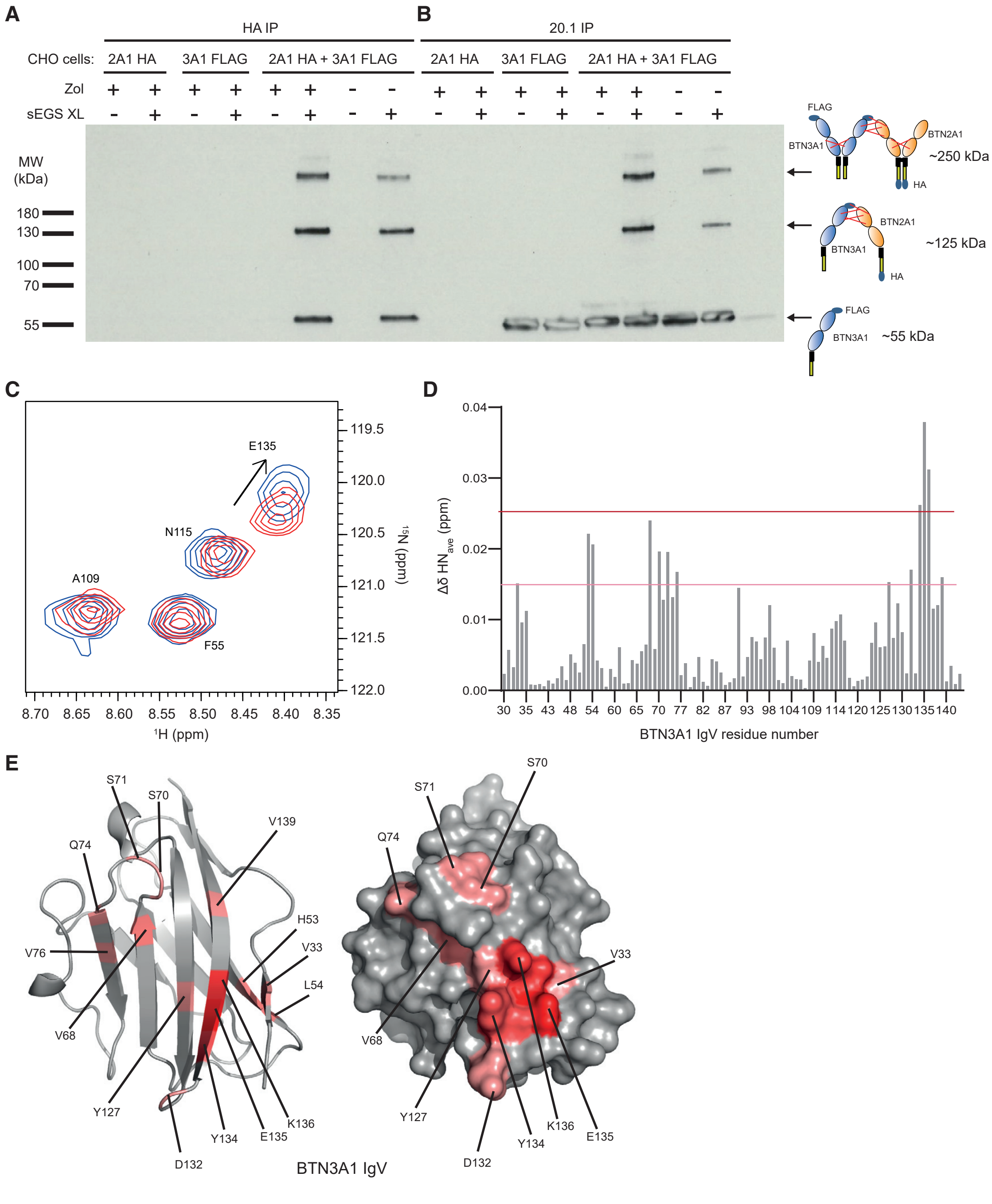

Figure 5. Cell-Surface Association of BTN2A1 and BTN3A1 Proteins

(A) Anti-BTN2A1-HA immunoprecipitation, combined with anti-BTN3A1-FLAG western blot detection, following cell-surface cross-linking of CHO cells expressing BTN2A1-HA, FLAG-BTN3A1, or both.

(B) Anti-BTN3A1 IP (20.1 mAb) of the same lysate combined with anti-BTN3A1-FLAG detection.

For (A) and (B), likely monomeric or oligomeric species corresponding to appropriate molecular weight bands are indicated on the right-hand side. Data are representative of four independent experiments.

(C) NMR chemical-shift perturbations (CSPs) in selected residues in ^{1}H-^{15}N-labeled BTN3A1 IgV (100 μM) following addition of BTN2A1 IgV (100 μM).

(D) Graph of chemical shift versus residue number in BTN3A1 IgV. Threshold levels for significant CSPs are indicated by horizontal lines.

(E) Mapping of residues whose amide resonances undergo CSPs on the surface of BTN3A1 IgV domain, showing clustering in the CFG face of the domain. Residues are colored in relation to the size of their CSPs, using the thresholds indicated in (D).

See also Figure S5.

BTN2A1 monomer and BTN3A1 monomer (125 kDa expected MW). A second represented a considerably larger cross-linked species (exceeding the weight of the 180 kDa marker), most likely equivalent to one BTN2A1 homodimer and one BTN3A1 homodimer (~250 kDa) (Figure 5A). In addition, both bands were also detected when the BTN3A1-specific mAb 20.1 was used for the initial immunoprecipitation step (Figure 5B), including when polyclonal anti-BTN2A1 antibody was used for WB detection (Figure S5). Incubation of CHO transductants with Zol was also used to assess if the presence of cross-linked BTN2A1-BTN3A1 species was dependent upon P-Ag levels (Figures 5A, 5B, and S5A). Of note, both higher-MW BTN2A1-BTN3A1 bands were detected in the presence and absence of Zol (Figures 5A, 5B, and S5A), indicating BTN2A1 association with BTN3A1 occurs constitutively.

To investigate whether BTN2A1-BTN3A1 association involved IgV-IgV domain interactions, we expressed and purified ^{1}H-^{15}N-labeled BTN3A1 IgV in *E. coli* and performed ^{1}H-^{15}N heteronuclear single quantum coherence (HSQC) spectroscopy in the absence and presence of an equimolar amount of naturally labeled *E. coli*-expressed BTN2A1 IgV (Figures 5C–5E and S5B). Analyses were facilitated by our previous assignment of all amide residues of the BTN3A1 IgV domain (Salim et al., 2017) and demonstrated small but significant chemical-shift perturbations (CSPs) in numerous BTN3A1 residues in the presence of BTN2A1 IgV (Figures 5C and 5D), indicating direct interaction. Mapping these CSPs onto the BTN3A1 IgV domain structure indicated the majority of these residues clustered around the CFG face of the BTN3A1 domain (Figure 5E). Importantly, this region of BTN3A1 or BTN3A2 (which share an identical IgV domain) has been highlighted as critical for P-Ag sensitization (Willcox et al., 2019), including specifically Y127, K136, and R73; notably, we show that both Y127 and K136 displayed detectable chemical shifts upon BTN2A1 binding, as did S70, S71, and Q74. In contrast, no CSPs were detected in similar experiments using ^{1}H-^{15}N-labeled BTN3A1 following addition of a control BTN family IgV domain (unlabeled BTNL3; Willcox et al., 2019) (Figures S5C and S5D). These results indicate that IgV-IgV domain interactions involving the CFG face of BTN3A1 or BTN3A2 contribute to BTN2A1-BTN3A1 association.

DISCUSSION

Here, we identified BTN2A1 as Factor X via a radiation hybrid approach that highlighted a critical ~580 kb region of Chr 6, in which we probed candidate genes that were retained in species that bear Vγ9Vδ2 T cells but were missing or non-conserved in mouse. BTN2A1-transduced rodent cells were specifically stained by Vγ9Vδ2 TCR tetramers, and crucially, BTN2A1 exhibited strong functional synergy with BTN3A1, restoring P-Ag sensing following co-transduction into mouse cells. Moreover, using SPR, we were able to demonstrate specific binding of BTN2A1 IgV domain to Vγ9Vδ2 TCRs. In addition, target cells transduced with BTN2A1 molecules incorporating single amino acid mutations that eliminated Vγ9Vδ2 TCR binding in SPR experiments failed to stimulate P-Ag-specific effector responses in Vγ9Vδ2 T cells. These findings not only establish BTN2A1 as the putative Factor X co-factor in P-Ag sensing but also highlight that its role as a direct ligand for the Vγ9Vδ2 TCR is essential to its ability to potentiate P-Ag sensing. Of note, Rigau et al. recently also identified BTN2A1 as a critical mediator of P-Ag sensing and a direct ligand for Vγ9^{+} TCRs using a TCR-tetramer staining and CRISPR-screen approach (Rigau et al., 2020).

Our results highlight the potential of radiation hybrids as a test system for identification of genomic regions controlling cellular phenotypes and function. The relatively simple screening scheme for identification of these regions by comparison of radiation hybrid transcriptomes facilitates the generation of custom-made radiation hybrids, which is an advantage over genetically defined radiation hybrid panels (Ross, 2001). Moreover, enabling rodent cells with capacity for P-Ag sensitization will not only help to understand Vγ9Vδ2 T cell function *in vitro* but also aid in establishing much-needed small animal models for the study of P-Ag-reactive cells. The De Libero group had shown (Kistowska, 2007) that Vγ9Vδ2 TCR transgenic mouse cells exhibit a block in thymic maturation, which can be overcome by administration of anti-CD3 antibody, suggesting a positive selection signal provided by species-specific molecules. We hypothesize that BTN2A1 and/or BTN3A1 are such molecules and aim to test whether *in vivo* expression of BTN2A1 and/or BTN3A1 enables Vγ9Vδ2 T cell maturation. If established, such a model would allow the determinants controlling γδ T cell responses and functionality in the emerging Vγ9Vδ2 T cell compartment to be studied and importantly would allow for development of small animal models for harnessing Vγ9Vδ2 T cells in pathological conditions such as cancer and infections with Vγ9Vδ2 T cell activating pathogens.

Establishment of direct binding experiments enabled us to probe the interaction mode of BTN2A1 with the Vγ9Vδ2 TCR. Our finding that TCR binding to BTN2A1 is solely dependent upon the TCR Vγ9 chain is entirely consistent with the finding that Vδ2 T cells expressing alternative non-Vγ9 Vγ regions are both insensitive to P-Ag and also adopt an adaptive-like biology fundamentally distinct from the innate-like features of Vγ9Vδ2 T cells (Davey et al., 2018). However, it was initially surprising, given that previous studies have highlighted the importance of multiple CDRs of both Vγ9 and Vδ2 TCR chains (including CDR3γ and CDR3δ) to Vγ9Vδ2-mediated P-Ag sensing (Wang et al., 2010); moreover, our mutagenesis studies provided additional confirmation of the importance of CDR3δ and CDR2δ residues for BTN2A1-stimulated P-Ag sensing. Furthermore, the exclusive focus of BTN2A1 on Vγ9 raised the question of whether the Vγ9Vδ2-BTN2A1 interaction mode was related to that of intestinal Vγ4 T cell recognition of BTNL3.8 (Willcox et al., 2019), which involves germline-encoded CDR2 and HV4 regions of the Vγ4 TCR chain and the CFG face of the BTNL3 IgV domain. Modeling and mutagenesis approaches confirmed a fundamental similarity with this binding mode, indicating both CDR2 and HV4 regions of the Vγ9 TCR chain, and residues in the CFG face of BTN2A1 were critical for recognition. These results highlight clear evolutionary conservation of the "superantigen-like" BTN or BTNL-γδ TCR interaction mode across different anatomical sites, which will no doubt be elucidated further by future structural analyses.

The oligomerisation state and interaction partners of BTN2A1 on the target cell surface are likely important factors in its mode of action. Specifically, we show that cell surface BTN2A1 is comprised predominantly of homodimers in transduced rodent cells and 293T cells. This is consistent with structural work that highlighted the potential of BTN3A1 to form IgC-IgC homodimers (Palakodeti et al., 2012), and indeed, our modeling

studies confirmed that BTN2A1 homodimers are likely to form highly equivalent IgC-IgC interactions. However, our results highlight the potential of BTN2A1 homodimer stabilization via an interchain disulphide-linkage involving a membrane-proximal cysteine residue absent in BTN2A2 and BTN3 molecules. Combined with our successful production of BTN2A1 IgV as a soluble functional monomeric domain, this suggests that BTN2A1 may form a "Y-shaped" dimer analogous to that proposed for BTN3A1 homo- and heterodimers (Palakodeti et al., 2012; Vantourout et al., 2018) and BTNL3.8 and BTNL1.6 heterodimers (Melandri et al., 2018; Willcox et al., 2019). Nevertheless, an important caveat is that although our studies suggest BTN2A1 preferentially homodimerizes even when co-expressed alongside BTN3A1, conservation of residues at the IgC interface means we cannot exclude non-disulphide-stabilized IgC-IgC-mediated heterodimeric interactions with other BTN molecules, including potentially BTN2A2 or, alternatively, BTN3A2 or BTN3A3 (Vantourout et al., 2018).

Importantly, we also establish a close association between BTN2A1 and BTN3A1 on target cells. While these results are consistent with those of Rigau et al., who determined co-localization of BTN2A1 and BTN3A1 to within the 10-nm resolution limit of FRET detection (Rigau et al., 2020), our immunoprecipitation approach employed a membrane-impermeable cross-linker featuring a 16-Å spacer arm, thereby suggestive of a close, possibly direct association at the cell surface. While importantly these experiments defined discrete, higher-MW species incorporating both BTN2A1 and BTN3A1, the requirement for chemical cross-linking to immunoprecipitate such complexes suggests the association is likely of relatively low affinity. Consistent with a direct interaction, our nuclear magnetic resonance (NMR) studies indicate that IgV-IgV interactions likely contribute to this *cis*-BTN2A1-BTN3A1 association and corroborate recent mutagenesis results (Willcox et al., 2019) that highlighted a critical role for residues on the CFG face of BTN3A1 or BTN3A2 IgV in P-Ag sensing. An interesting precedent for involvement of this CFG face in IgSF interactions in *cis* is provided by a recent study by Chaudhri and colleagues, who showed that PD-L1 may similarly utilize the CFG face of its IgV domain to mediate *cis*-interactions with B7.1 (Chaudhri et al., 2018). Notably, similar immunoprecipitation results were obtained in the presence or absence of Zol, which stimulates P-Ag accumulation in target cells, indicating that while likely essential for P-Ag sensing, BTN2A1-BTN3A1 association per se may not provide the critical molecular signal for Vγ9Vδ2 activation. Of relevance, the potential for BTN3A1 to heterodimerize with BTN3A2 or BTN3A3 and promote optimal P-Ag sensing (Vantourout et al., 2018), the identical IgV domain sequences of BTN3A1 and BTN3A2, and recent mutagenesis results on BTN3A1 and BTN3A2 (Willcox et al., 2019) are important considerations in interpreting these results and suggest that BTN2A1 IgV interactions with BTN3 could involve IgV domains of different BTN3 family members.

Collectively, our results and those of Rigau et al. (2020) revise current models of P-Ag sensing, many of which have previously focused on BTN3A1 as a "lone TCR ligand," and proposed either direct presentation of P-Ag by the IgV domain (Vavassori et al., 2013) or "inside-out" models whereby binding of P-Ag to the intracellular BTN3A1 B30.2 domain is transmitted in some way to the extracellular region of BTN3A1, creating a TCR binding site (Sandstrom et al., 2014; Wang et al., 2013). Importantly, our results do not exclude the possibility of direct TCR-BTN3A1 interactions, and indeed, recent mutagenesis of BTN3A1 and BTN3A2 (Willcox et al., 2019) could be interpreted as supporting Vγ9-BTN3A1 or BTN3A2 binding using a mode similar to both Vγ4-BTNL3 and Vγ9-BTN2A1. Nor can we discount the possibility of parallel and/or sequential interaction of Vγ9 TCR with BTN2A1 or BTN3A1 IgV domains. However, the unequivocal demonstration by our study and by Rigau et al. (2020) of direct Vγ9-BTN2A1 interaction, combined with lack of any compelling evidence for direct TCR-BTN3A1 or TCR-BTN3A2 interaction and finally our detection of direct BTN2A1-BTN3A1 complexes in this study, point to alternative possibilities. Taken together, these observations strongly suggest a composite ligand model of Vγ9Vδ2 recognition involving coordinate Vγ9-germline-mediated interaction with BTN2A1 alongside a CDR3-mediated interaction with a separate ligand(s). The identity of such a TCR ligand and its potential association partners at the cell surface is currently a focus of investigation. One possibility that cannot be excluded is that BTN3A1 is itself recognized in complex with BTN2A1 following P-Ag exposure, although our demonstration of constitutive BTN2A1-BTN3A1 association might argue against this; given that BTN2A1-BTN3A1 complexes occur in the absence of P-Ag, this would still require an inside-out mechanism to configure complexes for productive TCR-mediated recognition. Alternatively, it is tempting to speculate that BTN3A1 could function (potentially with facilitation by BTN3A2 or BTN3A3) to chaperone a critical additional Vγ9Vδ2 TCR ligand to the surface to be coordinately recognized as part of a BTN3A1-ligand complex in a CDR3-mediated fashion alongside BTN2A1. One prediction of this model is that such a CDR3-recognized ligand(s) is likely to be highly conserved between humans and rodents. Of note, instead of invoking direct BTN3A1 IgV interaction with the Vγ9Vδ2 TCR, this second model proposes BTN3A1 association with BTN2A1 as a mechanism of recruiting another ligand to the complex following P-Ag exposure; this would allow the Vγ9Vδ2 T cell compartment to continually survey BTN3A1-BTN2A1 complexes for the presence of a P-Ag-regulated ligand. In this context, alongside Vγ9 interaction with BTN2A1, such BTN3A1-BTN2A1 interactions most likely serve to spatially orientate BTN3A homo- or heterodimers and, following P-Ag exposure, an associated ligand, appropriately for TCR CDR3-mediated recognition. In this second composite ligand model, P-Ag binding to BTN3A1 B30.2 could regulate the strength of BTN3 association with such a ligand, and/or trafficking of such complexes to the cell surface. Further studies are required to clarify such mechanistic models, define structural features of the key interactions, address issues such as the role of BTN2A1 B30.2 domain, and establish relevance to other γδ T cell subsets, including the intestinal Vγ4 compartment.

In summary, we show that by acting as a direct ligand for the Vγ9Vδ2 TCR, BTN2A1 powerfully synergizes with BTN3A1 to potentiate P-Ag sensing. Vγ9Vδ2 T cells have to date been the primary focus of therapeutic development for γδ T cells. Understanding their mode of action should facilitate attempts to harness them therapeutically for either cell therapy or small molecule approaches.

STAR★METHODS

Detailed methods are provided in the online version of this paper and include the following:

- KEY RESOURCES TABLE
- LEAD CONTACT AND MATERIALS AVAILABILITY
- EXPERIMENTAL MODEL AND SUBJECT DETAILS
- METHOD DETAILS
  - Generation of radiation hybrids
  - RNAseq analysis of Radiation Hybrids
  - In vitro stimulation with human Vγ9Vδ2 TCR transductants
  - Expansion of primary human polyclonal Vγ9Vδ2 T cells
  - Generation of 293T *BTN2*$^{-/-}$ cell lines
  - Human IFNγ assay
  - Cloning and expression of BTN2A1, BTN2A2 or mutants
  - Generation of FLAG/HA tagged BTN3A1 and BTN2A1
  - Generation of Vγ9Vδ2 TCR (MOP) and mutant TCR chains
  - Soluble protein production
  - Flow cytometry/TCR tetramer staining
  - Surface plasmon resonance
  - Immunoprecipitation, surface biotinylation, and cross-linking
  - I-TASSER modeling of BTN2A1 and BTN2A2 ecto-domains
  - Modeling the BTN2A1-IgV/Vγ9 complex
  - Analysis of structural modeling and mutagenesis data
  - NMR
  - Software
- QUANTIFICATION AND STATISTICAL ANALYSIS
  - Statistical analyses
- DATA AND CODE AVAILABILITY

SUPPLEMENTAL INFORMATION

Supplemental Information can be found online at https://doi.org/10.1016/j.immuni.2020.02.014.

ACKNOWLEDGMENTS

We thank the University of Birmingham Protein Expression Facility for use of their equipment and Aravindan Viswanathan for help with generation of BTN2A1 and BTN2A2 CRISPR cell lines. This work was supported by the Wellcome Trust, United Kingdom (grants 099266/Z/12/Z and 099266/Z/12/A to B.E.W. supporting C.R.W., M.S., F.M., and C.B.); Deutsche Krebshilfe, Germany to T.H. (grant 70112079) and V.K. (grant 70112081) supporting D.P. and M.M.K.; Wilhelm–Sanderstiftung, Germany, grant 2013.907.2 to T.H. supporting M.M.K.; Deutsche Forschungsgemeinschaft grant HE2346/8-1 to T.H. supporting A.F., D.P., and M.M.K.; and SIRIC BRIO and the Ligue Nationale contre le Cancer, France (to J.D.-M. supporting V.P.). NMR studies were supported in part by the Wellcome Trust, United Kingdom (grant 208400/Z/17/Z to University of Birmingham), and we thank HWB-NMR staff at the University of Birmingham for providing open access to their Wellcome Trust-funded spectrometers. We gratefully acknowledge Adrian Hayday, Pierre Vantourout, and Fedor Berditchevski for useful discussions.

AUTHOR CONTRIBUTIONS

Conceptualization, T.H., M.M.K., C.R.W., and B.E.W.; Investigation, M.M.K., C.R.W., M.S., D.P., A.S.F., A. Noll, A. Nöhren, L.S., V.K., C.R.B., K.A.B., T.J.K., B.K., L.W., M.J., F.M., V.P., J.D.-M., R.A.G.C., and P.A.B.; Writing – Original Draft, B.E.W. and T.H.; Writing – Review & Editing, B.E.W., T.H., M.M.K., and C.R.W.; Visualization, M.M.K., C.R.W., T.H., B.E.W., and F.M.; Funding Acquisition, T.H. and B.E.W.; Supervision, T.H., B.E.W., C.R.W., and M.J.

DECLARATION OF INTERESTS

There are no competing interests to declare.

Received: January 20, 2020
Revised: February 18, 2020
Accepted: February 24, 2020
Published: March 9, 2020

REFERENCES

Boyden, L.M., Lewis, J.M., Barbee, S.D., Bas, A., Girardi, M., Hayday, A.C., Tigelaar, R.E., and Lifton, R.P. (2008). Skint1, the prototype of a newly identified immunoglobulin superfamily gene cluster, positively selects epidermal gammadelta T cells. Nat. Genet. *40*, 656–662.

Chaudhri, A., Xiao, Y., Klee, A.N., Wang, X., Zhu, B., and Freeman, G.J. (2018). PD-L1 Binds to B7-1 Only *In Cis* on the Same Cell Surface. Cancer Immunol. Res. *6*, 921–929.

Davey, M.S., Willcox, C.R., Hunter, S., Kasatskaya, S.A., Remmerswaal, E.B.M., Salim, M., Mohammed, F., Bemelman, F.J., Chudakov, D.M., Oo, Y.H., and Willcox, B.E. (2018). The human Vδ2$^+$ T-cell compartment comprises distinct innate-like Vγ9$^+$ and adaptive Vγ9$^-$ subsets. Nat. Commun. *9*, 1760.

Davodeau, F., Peyrat, M.A., Hallet, M.M., Gaschet, J., Houde, I., Vivien, R., Vie, H., and Bonneville, M. (1993). Close correlation between Daudi and mycobacterial antigen recognition by human gamma delta T cells and expression of V9JPC1 gamma/V2DJC delta-encoded T cell receptors. J. Immunol. *151*, 1214–1223.

Delfau, M.H., Hance, A.J., Lecossier, D., Vilmer, E., and Grandchamp, B. (1992). Restricted diversity of V gamma 9-JP rearrangements in unstimulated human gamma/delta T lymphocytes. Eur. J. Immunol. *22*, 2437–2443.

Di Marco Barros, R., Roberts, N.A., Dart, R.J., Vantourout, P., Jandke, A., Nussbaumer, O., Deban, L., Cipolat, S., Hart, R., Iannitto, M.L., et al. (2016). Epithelia Use Butyrophilin-like Molecules to Shape Organ-Specific γδ T Cell Compartments. Cell *167*, 203–218.

Fichtner, A.S., Karunakaran, M.M., Starick, L., Truman, R.W., and Herrmann, T. (2018). The Armadillo (*Dasypus novemcinctus*): A Witness but Not a Functional Example for the Emergence of the Butyrophilin 3/Vγ9Vδ2 System in Placental Mammals. Front. Immunol. *9*, 265.

Fichtner, A.S., Karunakaran, M.M., Gu, S., Boughter, C.T., Borowska, M.T., Starick, L., Noehren, A., Goebel, T.W., Adams, E.J., and Herrmann, T. (2020). Alpaca (Vicugna pacos), the first non-primate species with a phosphoantigen-reactive V-gamma-9 V-delta-2 T cell subset. Proc. Nat. Acad. Sci. USA. https://doi.org/10.1073/pnas.1909474117.

Gober, H.J., Kistowska, M., Angman, L., Jenö, P., Mori, L., and De Libero, G. (2003). Human T cell receptor gammadelta cells recognize endogenous mevalonate metabolites in tumor cells. J. Exp. Med. *197*, 163–168.

Halary, F., Pitard, V., Dlubek, D., Krzysiek, R., de la Salle, H., Merville, P., Dromer, C., Emilie, D., Moreau, J.F., and Déchanet-Merville, J. (2005). Shared reactivity of Vdelta2(neg) gammadelta T cells against cytomegalovirus-infected cells and tumor intestinal epithelial cells. J. Exp. Med. *201*, 1567–1578.

Harly, C., Guillaume, Y., Nedellec, S., Peigné, C.M., Mönkkönen, H., Mönkkönen, J., Li, J., Kuball, J., Adams, E.J., Netzer, S., et al. (2012). Key implication of CD277/butyrophilin-3 (BTN3A) in cellular stress sensing by a major human γδ T-cell subset. Blood *120*, 2269–2279.

Karunakaran, M.M., and Herrmann, T. (2014). The Vγ9Vδ2 T Cell Antigen Receptor and Butyrophilin-3 A1: Models of Interaction, the Possibility of Co-Evolution, and the Case of Dendritic Epidermal T Cells. Front. Immunol. *5*, 648.

Karunakaran, M.M., Göbel, T.W., Starick, L., Walter, L., and Herrmann, T. (2014). Vγ9 and Vδ2 T cell antigen receptor genes and butyrophilin 3 (BTN3)

emerged with placental mammals and are concomitantly preserved in selected species like alpaca (Vicugna pacos). Immunogenetics *66*, 243–254.

Kistowska, M. (2007). Antigen Recognition and Thymic Maturation of Human TCR Vγ9-Vδ2 Cells (Basel, Switzerland: PhD Basel University), p. 202.

Kunzmann, V., Bauer, E., Feurle, J., Weissinger, F., Tony, H.P., and Wilhelm, M. (2000). Stimulation of gammadelta T cells by aminobisphosphonates and induction of antiplasma cell activity in multiple myeloma. Blood *96*, 384–392.

Li, J.-Q. (2010). Modulating the expression of enzymes of isoprenoid synthesis: effects on Vgamma9Vdelta2 T cell activation and tumor cell growth. https://nbn-resolving.org/urn:nbn:de:bvb:20-opus-46388.

Melandri, D., Zlatareva, I., Chaleil, R.A.G., Dart, R.J., Chancellor, A., Nussbaumer, O., Polyakova, O., Roberts, N.A., Wesch, D., Kabelitz, D., et al. (2018). The γδTCR combines innate immunity with adaptive immunity by utilizing spatially distinct regions for agonist selection and antigen responsiveness. Nat. Immunol. *19*, 1352–1365.

Morita, C.T., Beckman, E.M., Bukowski, J.F., Tanaka, Y., Band, H., Bloom, B.R., Golan, D.E., and Brenner, M.B. (1995). Direct presentation of nonpeptide prenyl pyrophosphate antigens to human gamma delta T cells. Immunity *3*, 495–507.

Morita, C.T., Jin, C., Sarikonda, G., and Wang, H. (2007). Nonpeptide antigens, presentation mechanisms, and immunological memory of human Vgamma2Vdelta2 T cells: discriminating friend from foe through the recognition of prenyl pyrophosphate antigens. Immunol. Rev. *215*, 59–76.

Nguyen, K., Li, J., Puthenveetil, R., Lin, X., Poe, M.M., Hsiao, C.C., Vinogradova, O., and Wiemer, A.J. (2017). The butyrophilin 3A1 intracellular domain undergoes a conformational change involving the juxtamembrane region. FASEB J. *31*, 4697–4706.

Palakodeti, A., Sandstrom, A., Sundaresan, L., Harly, C., Nedellec, S., Olive, D., Scotet, E., Bonneville, M., and Adams, E.J. (2012). The molecular basis for modulation of human Vγ9Vδ2 T cell responses by CD277/butyrophilin-3 (BTN3A)-specific antibodies. J. Biol. Chem. *287*, 32780–32790.

Parker, C.M., Groh, V., Band, H., Porcelli, S.A., Morita, C., Fabbi, M., Glass, D., Strominger, J.L., and Brenner, M.B. (1990). Evidence for extrathymic changes in the T cell receptor gamma/delta repertoire. J. Exp. Med. *171*, 1597–1612.

Rhodes, D.A., Reith, W., and Trowsdale, J. (2016). Regulation of Immunity by Butyrophilins. Annu. Rev. Immunol. *34*, 151–172.

Riaño, F., Karunakaran, M.M., Starick, L., Li, J., Scholz, C.J., Kunzmann, V., Olive, D., Amslinger, S., and Herrmann, T. (2014). Vγ9Vδ2 TCR-activation by phosphorylated antigens requires butyrophilin 3 A1 (BTN3A1) and additional genes on human chromosome 6. Eur. J. Immunol. *44*, 2571–2576.

Rigau, M., Ostrouska, S., Fulford, T.S., Johnson, D.N., Woods, K., Ruan, Z., McWilliam, H.E.G., Hudson, C., Tutuka, C., Wheatley, A.K., et al. (2020). Butyrophilin 2A1 is essential for phosphoantigen reactivity by γδ T cells. Science *367*.

Ross, S.R. (2001). Commentary: phenotypic screening of radiation hybrid panels. Mamm. Genome *12*, 879–881.

Salim, M., Knowles, T.J., Baker, A.T., Davey, M.S., Jeeves, M., Sridhar, P., Wilkie, J., Willcox, C.R., Kadri, H., Taher, T.E., et al. (2017). BTN3A1 Discriminates γδ T Cell Phosphoantigens from Nonantigenic Small Molecules via a Conformational Sensor in Its B30.2 Domain. ACS Chem. Biol. *12*, 2631–2643.

Sambrook, J., and Russell, D.W. (2006). Calcium-phosphate-mediated Transfection of Eukaryotic Cells with Plasmid DNAs. CSH Protoc. *2006*.

Sanderson, S., and Shastri, N. (1994). LacZ inducible, antigen/MHC-specific T cell hybrids. Int. Immunol. *6*, 369–376.

Sandstrom, A., Peigné, C.M., Léger, A., Crooks, J.E., Konczak, F., Gesnel, M.C., Breathnach, R., Bonneville, M., Scotet, E., and Adams, E.J. (2014). The intracellular B30.2 domain of butyrophilin 3A1 binds phosphoantigens to mediate activation of human Vγ9Vδ2 T cells. Immunity *40*, 490–500.

Shen, L., Frencher, J., Huang, D., Wang, W., Yang, E., Chen, C.Y., Zhang, Z., Wang, R., Qaqish, A., Larsen, M.H., et al. (2019). Immunization of Vγ2Vδ2 T cells programs sustained effector memory responses that control tuberculosis in nonhuman primates. Proc. Natl. Acad. Sci. USA *116*, 6371–6378.

Silva-Santos, B., Mensurado, S., and Coffelt, S.B. (2019). γδ T cells: pleiotropic immune effectors with therapeutic potential in cancer. Nat. Rev. Cancer *19*, 392–404.

Smedley, D., Haider, S., Durinck, S., Pandini, L., Provero, P., Allen, J., Arnaiz, O., Awedh, M.H., Baldock, R., Barbiera, G., et al. (2015). The BioMart community portal: an innovative alternative to large, centralized data repositories. Nucleic Acids Res. *43*, W589–W598.

Soneoka, Y., Cannon, P.M., Ramsdale, E.E., Griffiths, J.C., Romano, G., Kingsman, S.M., and Kingsman, A.J. (1995). A transient three-plasmid expression system for the production of high titer retroviral vectors. Nucleic Acids Res. *23*, 628–633.

Starick, L., Riano, F., Karunakaran, M.M., Kunzmann, V., Li, J., Kreiss, M., Amslinger, S., Scotet, E., Olive, D., De Libero, G., and Herrmann, T. (2017). Butyrophilin 3A (BTN3A, CD277)-specific antibody 20.1 differentially activates Vγ9Vδ2 TCR clonotypes and interferes with phosphoantigen activation. Eur. J. Immunol. *47*, 982–992.

Vantourout, P., Laing, A., Woodward, M.J., Zlatareva, I., Apolonia, L., Jones, A.W., Snijders, A.P., Malim, M.H., and Hayday, A.C. (2018). Heteromeric interactions regulate butyrophilin (BTN) and BTN-like molecules governing γδ T cell biology. Proc. Natl. Acad. Sci. USA *115*, 1039–1044.

van Zundert, G.C.P., Rodrigues, J.P.G.L.M., Trellet, M., Schmitz, C., Kastritis, P.L., Karaca, E., Melquiond, A.S.J., van Dijk, M., de Vries, S.J., and Bonvin, A.M.J.J. (2016). The HADDOCK2.2 Web Server: User-Friendly Integrative Modeling of Biomolecular Complexes. J. Mol. Biol. *428*, 720–725.

Vavassori, S., Kumar, A., Wan, G.S., Ramanjaneyulu, G.S., Cavallari, M., El Daker, S., Beddoe, T., Theodossis, A., Williams, N.K., Gostick, E., et al. (2013). Butyrophilin 3A1 binds phosphorylated antigens and stimulates human γδ T cells. Nat. Immunol. *14*, 908–916.

Vranken, W.F., Boucher, W., Stevens, T.J., Fogh, R.H., Pajon, A., Llinas, M., Ulrich, E.L., Markley, J.L., Ionides, J., and Laue, E.D. (2005). The CCPN data model for NMR spectroscopy: development of a software pipeline. Proteins *59*, 687–696.

Wang, H., Fang, Z., and Morita, C.T. (2010). Vgamma2Vdelta2 T Cell Receptor recognition of prenyl pyrophosphates is dependent on all CDRs. J. Immunol. *184*, 6209–6222.

Wang, H., Henry, O., Distefano, M.D., Wang, Y.C., Räikkönen, J., Mönkkönen, J., Tanaka, Y., and Morita, C.T. (2013). Butyrophilin 3A1 plays an essential role in prenyl pyrophosphate stimulation of human Vγ2Vδ2 T cells. J. Immunol. *191*, 1029–1042.

Willcox, B.E., Gao, G.F., Wyer, J.R., Ladbury, J.E., Bell, J.I., Jakobsen, B.K., and van der Merwe, P.A. (1999). TCR binding to peptide-MHC stabilizes a flexible recognition interface. Immunity *10*, 357–365.

Willcox, C.R., Pitard, V., Netzer, S., Couzi, L., Salim, M., Silberzahn, T., Moreau, J.F., Hayday, A.C., Willcox, B.E., and Déchanet-Merville, J. (2012). Cytomegalovirus and tumor stress surveillance by binding of a human γδ T cell antigen receptor to endothelial protein C receptor. Nat. Immunol. *13*, 872–879.

Willcox, C.R., Vantourout, P., Salim, M., Zlatareva, I., Melandri, D., Zanardo, L., George, R., Kjaer, S., Jeeves, M., Mohammed, F., et al. (2019). Butyrophilin-like 3 Directly Binds a Human Vγ4+ T Cell Receptor Using a Modality Distinct from Clonally-Restricted Antigen. Immunity 51, 813–825.

Yang, J., and Zhang, Y. (2015). I-TASSER server: new development for protein structure and function predictions. Nucleic Acides Res. *43*, W174–W181.

STAR★METHODS

KEY RESOURCES TABLE

REAGENT or RESOURCE	SOURCE	IDENTIFIER
Antibodies		
Anti-huBTN3 (CD277) clone 103.2	Gift from Dr. Daniel Olive	N/A
Anti-huBTN3 (CD277) clone 20.1	Invitrogen	Cat# 14-2779-82; RRID: AB_467550
Anti-huBTN2A1 (1C7D)	MBL	Cat# W005-3
FITC anti-human Vδ2	BD Biosciences	Cat# 562088; RRID: AB_10892810
F(ab') Donkey anti mouse IgG (H+L) R-PE	Jackson Immunoresearch	Cat# 715-116-151; RRID: AB_2340799
mIgG1,κ isotype clone p3.6.2.81	eBiosciences	Cat#16-4714-85; RRID: AB_470162
mIgG2a,κ isotype clone-eBM2a	eBiosciences	Cat#16-4724-85; RRID: AB_470165
Anti-HA.11 epitope tag affinity matrix (clone 16B12)	Biolegend	Cat#900801; RRID: AB_2564999
Purified anti-DYKDDDDK (FLAG) tag antibody (clone L5)	Biolegend	Cat#637301; RRID: AB_1134266
Anti-HA.11 epitope tag antibody, FITC labeled (Clone 16B12)	Biolegend	Cat#901507; RRID: AB_2565058
BTN2A1 rabbit polyclonal antibody	Sigma	Cat#HPA019208; RRID: AB_1845492
Goat anti-rabbit HRP	ThermoFisher	Cat#G21234; RRID: AB_2536530
Goat anti-rat HRP	ThermoFisher	Cat#A10549; RRID: AB_2534047
Purified mouse anti-human TCRγ/δ, clone 11F2	BD Biosciences	Cat# 347900; RRID: AB_400356
Anti-Vγ9 antibody, FITC (IMMU360)	Beckman Coulter	Cat#IM1463; RRID: AB_130871
Bacterial and Virus Strains		
NEB 5-alpha	NEB	Cat# C2987H
BL21 (DE3)	NEB	Cat# C2527H
Biological Samples		
BrHPP-expanded Vγ9Vδ2 T cells	This paper	N/A
Chemicals, Peptides, and Recombinant Proteins		
HMBPP	Sigma	Cat#95058
Zoledronate	Sigma	Cat#SML0223
rhIL-2	AiCuris	Ch.B.: ZA4621B/3
Phusion high fidelity DNA polymerase	ThermoFisher Scientific	Cat#F530S
IN-Fusion HD cloning Kit	TAKARA	Cat#639649
TOPO TA Cloning kit for sequencing	Invitrogen	Cat#450071
HAT Media Supplement (50x) Hybrid-Max	Sigma	Cat#H0262
HT Media Supplement (50x) Hybrid-Max	Sigma	Cat#H0137
PEG 1500	Roche	Cat# 10 783 641 001
Histopaque-1077	Sigma	Cat#10711
GeneArt CRISPR Nuclease (OFP reporter)	Invitrogen	Cat#A21174
GeneArt CRISPR Nuclease (CD4 enrichment)	Invitrogen	Cat#A21175
EcoRI	ThermoFisher Scientific	Cat#ER0271
NdeI	Roche	Cat# 11 040 227 001
BamHI	Roche	Cat# 10 567 604 001
BTN2A1 IgV	This paper	N/A
BTN2A2 IgV	This paper	N/A
Soluble T cell receptors (sTCRs)	Willcox et al., 2012; this paper	N/A
Streptavidin-HRP	ThermoFisher Scientific	Cat#21130
Streptavidin-PE conjugate	ThermoFisher Scientific	Cat#S866
Streptavidin-APC	ThermoFisher Scientific	Cat#S868
Sulfo-EGS crosslinker	ThermoFisher Scientific	Cat#21566

(*Continued on next page*)

Continued

REAGENT or RESOURCE	SOURCE	IDENTIFIER
EZ-link Sulfo-NHS-LC biotin	ThermoFisher Scientific	Cat#21335
Iodoacetamide	Sigma	Cat#I6125
Critical Commercial Assays		
IL-2 mouse uncoated ELISA kit	Invitrogen	Cat # 88-7024-88
IFN gamma Human uncoated ELISA kit	Invitrogen	Cat # 88-7316-88
Deposited Data		
RNA-seq dataset of radiation hybrid clones filtered for transcribed human genes	Mendeley Data	https://doi.org/10.17632/ny6bxn4y9s.1
Experimental Models: Cell Lines		
293T	DSMZ	Cat#ACC 635; RRID: CVCL_0063
CHO (CHO-K1)	ATCC	Cat#CCL-61; RRID: CVCL_0214
CHO human Chromosome 6	Coriell Institute for Medical research	GM11580; RRID: CVCL_V287
BW36 gal (BW)	Dr. Nilabh Shastri Lab; Sanderson and Shastri, 1994	N/A
53/4 hybridoma Vγ9Vδ2 - MOP TCR	Starick et al., 2017	N/A
A23 Thymidine kinase negative Hamster fibroblast (HAT sensitive)	Dr. Carol Stocking Lab	N/A
BW 58C-CD28+	Harly et al., 2012	N/A
Oligonucleotides		
Primer and CRISPR Sequences in Tables S1 and S2	N/A	N/A
Recombinant DNA		
pMIM	gift from Dario Vignali	Addgene # 52114
pMIG II	gift from Dario Vignali	Addgene # 52107
pIZ	Gift from Dr. Ingolf Berberich	N/A
pIH	Gift from Dr. Ingolf Berberich	N/A
pIH-FLAG	This paper	N/A
pET23a	Merck Millipore	Cat# 69745-3
pMT/BiP/V5-HisB	Invitrogen	Cat# V413020
BTN2A1 IgV in pET23a (wild type and mutants)	This paper	N/A
BTN2A2 IgV in pET23a	This paper	N/A
BTN3A1 IgV in pET23a	(Salim et al., 2017)	N/A
Human and mouse γδTCRs in pMT/BiP/V5-HisB	This paper; Willcox et al., 2012	N/A
Software and Algorithms		
FlowJo version 10	FlowJo	https://flowjo.co/
PyMOL version 2.0.7	Schrodinger	https://pymol.org/2/
GraphPad Prism version 8.0.2	GraphPad Software	https://www.graphpad.com
BIAevaluation	GE Healthcare	https://www.gelifesciences.com/en/gb/shop/protein-analysis/spr-label-free-analysis
Origin 2015	OriginLab	https://www.originlab.com/
CRISPR design tool	Invitrogen	N/A
ZHANG LAB	Vantourout et al., 2018	https://zlab.bio/guide-design-resources
CRISPR RGEN tools	This paper	http://www.rgenome.net/
Other		
Sensor Chip CM5	GE Healthcare	Cat#29149604
Sensor Chip NTA	GE Healthcare	Cat#BR100407
HBS-P	GE Healthcare	Cat#BR100368
HBS-EP	GE Healthcare	Cat#BR100188
streptavidin	Sigma	Cat#S4622

LEAD CONTACT AND MATERIALS AVAILABILITY

Further information and requests for resources and reagents should be directed to and will be fulfilled by the Lead Contact, Benjamin E. Willcox (b.willcox@bham.ac.uk). Reagents generated in this study are available on request from the Lead Contact with a completed Materials Transfer Agreement.

EXPERIMENTAL MODEL AND SUBJECT DETAILS

CHO, CHO-chr6, BW, A23, 53/4 hybridoma TCR transductants, and radiation hybrids (CHO Chr6 – rodent fusion hybrids) were cultured with RPMI (GIBCO) supplemented with 10% FCS, 1 mM sodium pyruvate, 2.05 mM glutamine, 0.1 mM nonessential amino acids, 50 μM β-mercaptoethanol, penicillin (100 U/mL) and streptomycin (100 U/mL). Peripheral blood mononuclear cells isolated from healthy volunteers were also maintained as above with or without rhIL-2 (Novartis Pharma). 293T cells were maintained in DMEM (GIBCO) supplemented with 10% FCS.

METHOD DETAILS

Generation of radiation hybrids

CHO Chr 6 (10^6 or 10^7 cells) were irradiated at Faxitron CP160 (program 160 kV, 6.3 mA, 300 Gy: 60 min, 100 Gy: 20 min). The irradiated cells and fusion partner (BW or A23) were mixed at 1:1 or 1:3 ratio (irradiated cell:fusion partner) and centrifuged at 461 g for 5 min at RT. The cell pellet was gently tapped and 1 mL PEG1500 was added slowly over a minute with gentle mixing in a prewarmed water-bath. After addition of PEG, cells were resuspended in 50 mL warm serum free RPMI and incubated for 30 min, followed by centrifugation at 461 g for 5 min and careful resuspension in RPMI supplemented with 10% FCS at 10^4 cells/mL. The cell suspension was seeded in 96 well plate flat bottom (A23 fused) or round bottom (BW fused) plates in 100 μl per well. On the following day, 100 μl of 2X HAT was added and cells were selected for two weeks. The selected clones were supplemented with HT medium and further seeded at limiting dilutions to obtain single cell clones which were tested for P-Ag mediated activation of our Vγ9Vδ2 TCR (MOP) transductants. P-Ag presentation capable and incapable clones were PCR characterized for human Chr 6 regions with primers listed in Table S1.

RNAseq analysis of Radiation Hybrids

Knowing the differences in antigen presentation of the various radiation hybrid cell lines, we performed RNA seq to identify those human Chr 6-encoded genes that are expressed in each hybrid line. Cells were stored in TRIzol Reagent (Invitrogen) and total RNA was extracted. Sequencing libraries were produced with an Illumina Truseq RNA preparation kit as described by the supplier's protocol and were sequenced with an Illumina HiSeq4000. Sequence reads were mapped to the human genome (hg38) with STAR (version STAR_2.50a) and read counts of gene transcripts were determined using gtf file Homo_sapiens.GRCH38.84.gtf and featureCount (v1.5.0-p1). Cell lines were then compared for presence, i.e., expression, of human Chr 6 genes. To filter out reads descending from mouse or hamster cells, all fastq-files were initially mapped against the mouse genome (*Mus musculus*, version GRCm38) using STAR and the corresponding Gene Transfer Format (gtf) file (version 87). Unmapped reads and those exhibiting more than two mismatches were selected and mapped against the Chinese hamster genome (*Cricelulus griseus*, version 1). The corresponding gtf-file was downloaded from the pre-Ensembl ftp site (Cricetulus_griseus.CriGri_1.pre.gtf). Afterward, all unmapped reads and those containing more than two mismatches were again selected to finally map against the human genome (version hg38; gtf-file version 84). Only reads showing maximally one mismatch were considered as true. With the help of featureCounts, mapped reads were assigned to genomic features using the above mentioned gtf-files. The results were summarized within an Excel-file. Further gene information were extracted from BioMart (Ensembl Genes 84; (Smedley et al., 2015).

In vitro stimulation with human Vγ9Vδ2 TCR transductants

For *in vitro* stimulations, 10^4 CHO or 293T cells were seeded on day 1 with 50 μL RPMI or DMEM in a 96 well flat bottom cell culture plate and cultured over-night. On day 2, 50 μL of 5x10^4 - 53/4 hybridoma cells expressing the human MOP Vγ9Vδ2 TCR and 100 μL of appropriate stimulant such as HMBPP, Zol, or 20.1 mAb were added to the culture and incubated for 22 h. After overnight incubation, the activation of TCR transductants was analyzed by measurement of mouse IL-2 from the supernatants of the co-cultures by ELISA (Invitrogen) as per manufacturer's protocol.

Expansion of primary human polyclonal Vγ9Vδ2 T cells

Fresh peripheral blood mononuclear cells (PBMCs) were isolated from healthy volunteers after obtained written informed consent in accordance with the Declaration of Helsinki and approval by the University of Würzburg institutional review board. Whole blood was layered over the Histopaque-1077 in a 50 mL falcon tube and centrifuged at 400 g for 30 min at room temperature (RT) with no acceleration and brakes. After centrifugation, the opaque interface containing PBMCs were aspirated and washed twice at 461 g for 5 min at RT. Vγ9Vδ2 T cells were expanded by cultivation of PBMCs with RPMI containing 10% FACS, 1 μM BrHPP and recombinant

human IL-2 100 IU/mL (Novartis Pharma) in 10^6 cells/mL density in a 96 well U bottom plate for 10 days with 100 µL per well. After 10 days, cells were pooled and washed twice and cultivated to rest without rhIL- 2 at 10^6 cells/mL density in a 6-well plate. After three days, rested cells were subjected for further experiments.

Generation of 293T *BTN2*$^{-/-}$ cell lines

BTN2A1 and *BTN2A2* genes were disrupted in 293T cells using CRISPR. The CRISPR sequencing targeting functional *BTN2A* genes were designed with the help of online tools mentioned in the table (software section) and sequences were cloned into GeneArt CRISPR Nuclease vector as per manufacturer's instructions. On day1, 1.5 × 10^6 293T cells were seeded in a 6 cm cell culture plate with DMEM medium without pyruvate (10% FCS). On day 2, cells were transfected with 5 µg of BTN2A-IgV_CRISPR cloned GeneArt CRISPR Nuclease (CD4 enrichment) Vector or BTN2A1_49FCRISPR cloned GeneArt CRISPR Nuclease (OFP Reporter) Vector or BTN2A2_343CRISPR cloned GeneArt CRISPR Nuclease (CD4 enrichment) Vector in a calcium-phosphate dependent method (Sambrook and Russell, 2006) (CRISPR sequences are provided in Table S2). 48 h post transfection, the highest reporter expressing (top 3%) cells were sorted and seeded at 1 cell/200 µL medium/well in 96 well plate flat bottom cell culture plate and cultivated till single cell derived clones were visible. Such clones were tested for their capacity to stimulate our 53/4 hybridoma human Vγ9Vδ2 TCR (MOP) TCR transductants in the presence of 1 µM HMBPP. The clones which exhibited loss of function were subjected to DNA isolation, followed by PCR for the amplification of genetic loci targeted by CRISPR sequences with appropriate genomic primers (Table S2) complementary to flanking regions of CRISPR target site. The PCR products were cloned into TOPO-TA vector (Invitrogen) and the TOPO-TA clones were analyzed by sequencing for the presence of in/del mutations resulting in loss of gene mutation as shown below. 293T *BTN2*$^{-/-}$ cell line harbor *BTN2A1* alleles with 10 and 16 nucleotide deletion, *BTN2A2* alleles with 1 and 10 nucleotide deletions; 293T *BTN2A1*$^{-/-}$ harbors *BTN2A1* alleles with 1 nucleotide addition and 10 nucleotide deletion; 293T *BTN2A2*−/− harbors *BTN2A2* alleles with 1 nucleotide deletion.

CRISPR target sites and allelic phenotypes

1a) *BTN2*−/− allelic phenotype

BTN2A1 IgV CRISPR target site

```
2A1IgV      GCAGTGTTTGTGTATAAAGGTGGCAGAGAGAGAACAGAGGAGCAGATGGAGGAGT 55
2A1allele1  GCAGTGTTT----------GTGGCAGAGAGAGAACAGAGGAGCAGATGGAGGAGT 45
2A1allele2  GCA----------------GTGGCAGAGAGAGAACAGAGGAGCAGATGGAGGAGT 39
            ***                ************************************
```

1b) *BTN2A2* IgV CRISPR target site

```
2A2IgV      GCAGTGTTTGTGTATAAGGGTGGGAGAGAGAGAACAGAGGAGCAGATGGAGGAGT 55
2A2allele1  GCAGTGTTTGTGTATA-GGGTGGGAGAGAGAGAACAGAGGAGCAGATGGAGGAGT 54
2A2allele2  GCAGTGTTTG----------TGGGAGAGAGAGAACAGAGGAGCAGATGGAGGAGT 45
            **********          ***********************************
```

2) *BTN2A1*−/− allelic phenotype

```
2A1-49F     GGACTAGGCTCTAAGCCCCTCATTTCAATGAGGGGCCATGAA-GACGGGGGCATCCGGC 59
Allele1     GGACTAGGCTCTAAGCCCCTCATTTCAATGAGGGGCCATGAAGGACGGGGGCATCCGGC 59
Allele2     GGACTAGGCTCTAAGCCCCTCATTTCAATGAGGGG--------------GGCATCCGGC 45
            ***********************************              **********
```

3) *BTN2A2*−/− allelic phenotype

```
2A2        CAAGAAGGCAGGTCCTACGATGAGGCCATCCTACGCC-TCGTGGTGGCA 48
Allele     CAAGAAGGCAGGTCCTACGATGAGGCCATCCTACGCCCTCGTGGTGGCA 49
           ************************************* ***********
```

Human IFNγ assay

293T or 293T *BTN2*$^{-/-}$ cells were seeded overnight in triplicates at 2 × 10^4 cells/well in 100 µL DMEM containing 10% FCS in a 96 well flat bottom cell culture plate with or without 25 µM Zol. Next day, DMEM with/without Zol was aspirated and cells were washed twice with PBS at RT and 2 × 10^4 expanded Vγ9Vδ2 T cells/well in 100µL RPMI was added and cocultured for 4 h. After 4 h, supernatants were collected and frozen at −20°C, until cytokine assay was performed with human IFNγ ELISA kit (Invitrogen) was used according to the manufacturer's instructions.

Cloning and expression of BTN2A1, BTN2A2 or mutants

The full length human *BTN2A1* and *BTN2A2* were amplified from the cDNA obtained from 293T cells with the help of pMIM-BTN2A1/2Fwd and pMIM-BTN2A1/2Rev primers (see Table S2) using Phusion DNA polymerase (Thermo Scientific). The amplified BTN2A1 or BTN2A2 PCR products were cloned into EcoRI & BamHI digested pMIM or pMIG II vector via In-Fusion HD cloning kit (Takara). BTN2A1 mutants were generated by fusion of two PCR products obtaining from pMIM-BTN2A1Fwd/Mutant-Rev and Mutant-Fwd/pMIM- BTN2A1/2Rev and cloned as above. Such cloned BTN2 genes and their corresponding mutants were expressed in target cells through retroviral transduction (Soneoka et al., 1995).

Generation of FLAG/HA tagged BTN3A1 and BTN2A1

For the N terminus FLAG or HA tagged proteins, FLAG or HA tag was inserted into the BamHI+EcoRI digested pIH or pIZ vector back bone with digested FLAG/HA Fwd and Rev oligonucleotide sequences with appropriate restriction sites and linker sequences flanking the FLAG/HA tag sequences. BTN3A1 and BTN2A1 full length amplicons without leader peptide were amplified either cDNA or above mentioned pMIM-BTN2A1 as template. BTN2A1-HA tagged constructed amplified with pMIM-BTN2A1 as template and pIZ-BTN2A1Fwd and BTN2A1- HA-Rev primers was cloned into pIZ vector backbone via In-Fusion-HD cloning as per manufacturer's protocol.

Generation of Vγ9Vδ2 TCR (MOP) and mutant TCR chains

Vγ9Vδ2 TCR (MOP), Vδ2-R51A and Vδ2-CDR3 deletion mutant (ΔCDR3: CDR3δ sequence CACD———YTDKLIF) TCR chains were generated as reported earlier (Li, 2010; Starick et al., 2017). Vγ9-E70A, -E70R and -E70K mutants were generated by fusion of two PCR products amplified by MOP- Vγ9 Fwd/Mut-Rev and Mut-Fwd/ MOP-Vγ9-Rev primers with pEGN-MOP-Vγ9 as template using Phusion polymerase. Such generated wild type TCR chains and mutant TCR chains were expressed in 53/4 hybridoma cells by retroviral transduction (Soneoka et al., 1995).

Soluble protein production

cDNA encoding wild type BTN2A1 (S27 to V142) or BTN2A2 IgV domains (S31 to V146), or BTN2A1 IgV incorporating the described mutations, were generated as gblocks (Integrated DNA Technologies) including the sequence for a C-terminal 6x Histidine tag and cloned into the pET23a expression vector (Novagen). Proteins were overexpressed, purified and refolded as described (Willcox et al., 2019). BTN2A1 and BTN2A2 IgV domains were refolded by dilution in 100 mM Tris, 400 mM L-Arginine-HCl, 2 mM EDTA, 6.8 mM cystamine, 2.7 mM cysteamine, 0.1 mM PMSF, pH 8, overnight at 4°C. The refolding mixture was concentrated and purified by size exclusion chromatography on a Superdex-200 column (GE Healthcare) pre-equilibrated with 20 mM Tris, 150 mM NaCl, pH 8, or 20 mM Na3PO4 pH 7.4 buffer, or PBS. BTN3A1 IgV was expressed, refolded, and purified as described (Salim et al., 2017). Soluble γδ TCRs were generated in *Drosophila* S2 cells and purified by nickel chromatography as previously described (Willcox et al., 2012). TCRs were then biotinylated via a C-terminal BirA tag.

Flow cytometry/TCR tetramer staining

Flow cytometry staining of the samples were performed with the below mentioned antibodies and samples were measured on FACSCalibur or LSRII flow cytometer (BD). The expression of BTN3A1 and BTN2A1 were detected with anti-huBTN3 (CD277) clone 103.2 (gift from David Olive) and anti-huBTN2A1 clone 1C7D (MBL), followed by secondary antibody F(ab') Donkey anti mouse IgG (H+L) R-PE (Jackson Immunoresearch). mIgG1,κ isotype clone p3.6.2.81 (eBiosciences) and mIgG2a,κ isotype clone-eBM2a (eBiosciences) were used a isotype controls and were detected by above mentioned secondary antibody. N-terminal HA-tagged BTN2A1 or BTN2A2 were detected using anti-HA-FITC (Biolegend). PBMC expanded human Vγ9Vδ2 T cells were detected with FITC- conjugated anti-human Vδ2 (BD Biosciences). Biotinylated soluble Vγ9Vδ2 TCRs were tetramerized by the addition of Streptavidin-PE conjugate (ThermoFisher Scientific) at room temperature, and 1-2μg of tetramer used to stain 10^5 cells at 4°C.

Surface plasmon resonance

SPR was performed as previously described (Willcox et al., 1999) on a BIAcore3000 using streptavidin-coated CM5 chips and HBS-EP buffer (GE Healthcare). Biotinylated Vγ9 TCRs, and control Vγ2, or Vγ4 TCRs (2000-3000 RU), were captured on the Streptavidin chip. Analyte concentrations ranged from 1-200 μM.

Immunoprecipitation, surface biotinylation, and crosslinking

293T cells in which the *BTN2A1* and *BTN2A2* loci have been functionally inactivated (293T *BTN2*$^{-/-}$ cells), or CHO CD80$^+$ cells, were transduced to overexpress HA-tagged BTN2A1 or BTN2A2, or BTN3A1, as indicated. Cells were surface biotinylated using EZ-Link Sulfo NHS-LC-biotin (ThermoFisher, 0.8mg/mL in PBS) for 30 min on ice, quenched with 20mM Tris pH 7.5 for 5 min, washed in TBS, and lysed in lysis buffer containing 1% NP40 in 20mM Tris pH 7.5, 150mM NaCl ± 10mM iodoacetamide (Sigma). HA-tagged BTN2A1 or BTN2A2 was immunoprecipitated using anti-HA resin (BioLegend). Immunoprecipitations were washed in lysis buffer and eluted in nonreducing (NR) or reducing (R) SDS sample buffer and boiled, or incubated at 37C for 5 min before separation on 4%–20% SDS-PAGE gels (BioRad). Proteins were transferred to PVDF using the BioRad TransBlot Turbo system, blocked in 3% BSA, then incubated with streptavidin-HRP (Thermo). To investigate potential association of BTN2A1 and BTN3A1 at the cell surface, CHO cells overexpressing BTN2A1-HA, FLAG-BTN3A1, or both, were treated with the soluble, membrane-impermeable crosslinker sulfo-EGS (ThermoFisher) at 0.5mM in PBS, at 4°C for 2 h. Following this, the reaction was quenched by addition of Tris pH 7.5 to 20mM. Cells were washed in TBS and lysed in 1% NP40 lysis buffer. After centrifugation to remove insoluble material, immunoprecipitation was carried out using anti-HA resin or 20.1 antibody bound to protein A Sepharose (GE Healthcare). Immunoprecipitates were run on duplicate 4%–20% gels (BioRad) and blotted with anti-BTN2A1 or anti-FLAG antibodies.

I-TASSER modeling of BTN2A1 and BTN2A2 ectodomains

The ectodomain structures of BTN2A1 (residues Q29-A248) and BTN2A2 (residues Q33-M265), were generated using the I-TASSER (Iterative Threading ASSEmbly Refinement) server (Yang and Zhang, 2015). Briefly, the target sequences were initially threaded

through the Protein Data Bank (PDB) library by LOMETS2, an online meta- threading server system for template-based protein prediction. Continuous fragments were excised from LOMETS2 alignments and structurally reassembled by replica-exchange Monte Carlo simulations. The simulation trajectories were then grouped and used as the initial state for second round I-TASSER assembly simulations. Finally, lowest energy structural models were identified and refined by fragment-guided molecular dynamic simulations to improve hydrogen-bonding contacts and omit steric clashes. Models were ranked based on their I-TASSER confidence (C) score (range -5 to +2 with a higher score correlating with a higher confidence model).

Modeling the BTN2A1-IgV/Vγ9 complex

The BTN2A1-IgV/Vγ9 complex was modeled with HADDOCK (van Zundert et al., 2016). BTN2A1 residues (R65, K79, R124, Y126 and E135) were classified as active in Vγ9 binding based upon the results of SPR binding experiments. 'Passively involved' residues were selected automatically. Vγ9 residues (R20, D72, E70 and E76) selected for use as ambiguous interaction restraints to drive the docking process with BTN2A1 were predicted from an initial homology model (generated by superimposing BTN2A1-IgV and Vγ9 onto the previously published BTNL3-IgV/Vγ4 complex model (Melandri et al., 2018).

Analysis of structural modeling and mutagenesis data

R65 (located in the IgV domain of BTN2A1) forms a salt bridge interaction with E76 (in the Vγ9 TCR chain). This interaction is likely to be abolished by introducing Ala at this position in BTN2A1 IgV domain (R65A), consistent with abrogation of binding by the R65A mutant. The hydroxyl group of S72 (BTN2A1) is in close proximity to V58 (TCR). Juxtaposition of this polar residue (S72) with a hydrophobic residue (V58) is likely to be energetically unfavorable for binding in this region. By substituting an Ala (ie a non-polar residue) at this position, the S72A mutation is likely to introduce hydrophobic interactions with V58, consistent with enhanced binding compared to wild-type (11-15μM (S72A) versus 50μM (Wild-type)).

K79A leads to reduced binding to TCR (100μM). K79 forms a salt bridge interaction with E76 (TCR). Change to Ala will result in loss of this interaction consistent with a reduction in binding affinity. The fact that binding is not totally abolished suggests that this interaction is a not a major contributor to the binding energy. Note however that E76 also contacts R65 (see above).

R124A mutation in BTN2A1 abolishes binding to TCR. The HADDOCK model suggests that R124 forms a salt bridge interaction with E70 (Vγ9-IgV TCR) and a hydrogen bonding interaction with the hydroxyl group of T83. These interactions will be lost when introducing an Ala at this position.

Y126A abolishes binding to TCR. Y126 forms multiple hydrophobic stacking interactions with I74 (HV4 region of Vg9-IgV). In addition, the hydroxyl group of Y126 forms a hydrogen bonding interaction with T77. These will be lost upon alanine substitution.

Although Y133A is located at the interface with Vγ9, it does not mediate interactions with Vγ9 TCR residues and hence it is unsurprising that substitution to alanine does not affect binding affinity. E135 forms a salt bridge interaction with R20 (TCR). Substitution to Ala will result in a loss of this interaction, and consistent with this, E135A mutation abolishes binding to the TCR.

NMR

HSQC experiments were performed at 298K on 600MHz Bruker Avance III spectrometer equipped with a 5 mm TCI cryogenically cooled triple resonance probe. Spectra were acquired using 100 μM ^{1}H-^{15}N-labeled BTN3A1. Experiments were processed using Topspin 3.2 (Bruker). All analysis was performed using CCPN Analysis (Vranken et al., 2005). For analysis of BTN3A1/BTN2A1 interaction, the final concentration of each protein was 100μM, and a threshold of 0.015 weighted average ppm difference was used as a cut-off to identify chemical shift perturbations in BTN3A1 residues upon BTN2A1 addition.

Software

Structural figures were generated in PyMOL (version 2.0.7; Schrodinger, LLC). SPR data was analyzed in BIAevaluation (GE Healthcare) and Origin 2015 (OriginLab).

QUANTIFICATION AND STATISTICAL ANALYSIS

Statistical analyses

Stimulation data and transcript visualization in Figures 1 and S1A were calculated and depicted using GraphPad Prism. Differences between transduced and untransduced cells were tested using 2-way ANOVA and unpaired multiple t test using the Holm-Sidak method.

DATA AND CODE AVAILABILITY

The RNaseq data of radiation hybrid clones filtered for transcribed human genes are available at Mendeley data https://doi.org/10.17632/ny6bxn4y9s.1

Immunity, Volume *52*

Supplemental Information

Butyrophilin-2A1 Directly Binds Germline-Encoded Regions of the Vγ9Vδ2 TCR and Is Essential for Phosphoantigen Sensing

Mohindar M. Karunakaran, Carrie R. Willcox, Mahboob Salim, Daniel Paletta, Alina S. Fichtner, Angela Noll, Lisa Starick, Anna Nöhren, Charlotte R. Begley, Katie A. Berwick, Raphaël A.G. Chaleil, Vincent Pitard, Julie Déchanet-Merville, Paul A. Bates, Brigitte Kimmel, Timothy J. Knowles, Volker Kunzmann, Lutz Walter, Mark Jeeves, Fiyaz Mohammed, Benjamin E. Willcox, and Thomas Herrmann

Immunity, Volume *52*

Supplemental Information

Butyrophilin-2A1 Directly Binds Germline-Encoded Regions of the Vγ9Vδ2 TCR and Is Essential for Phosphoantigen Sensing

Mohindar M. Karunakaran, Carrie R. Willcox, Mahboob Salim, Daniel Paletta, Alina S. Fichtner, Angela Noll, Lisa Starick, Anna Nöhren, Charlotte R. Begley, Katie A. Berwick, Raphaël A.G. Chaleil, Vincent Pitard, Julie Dechanet-Merville, Paul A. Bates, Brigitte Kimmel, Timothy J. Knowles, Volker Kunzmann, Lutz Walter, Mark Jeeves, Fiyaz Mohammed, Benjamin E. Willcox, and Thomas Herrmann

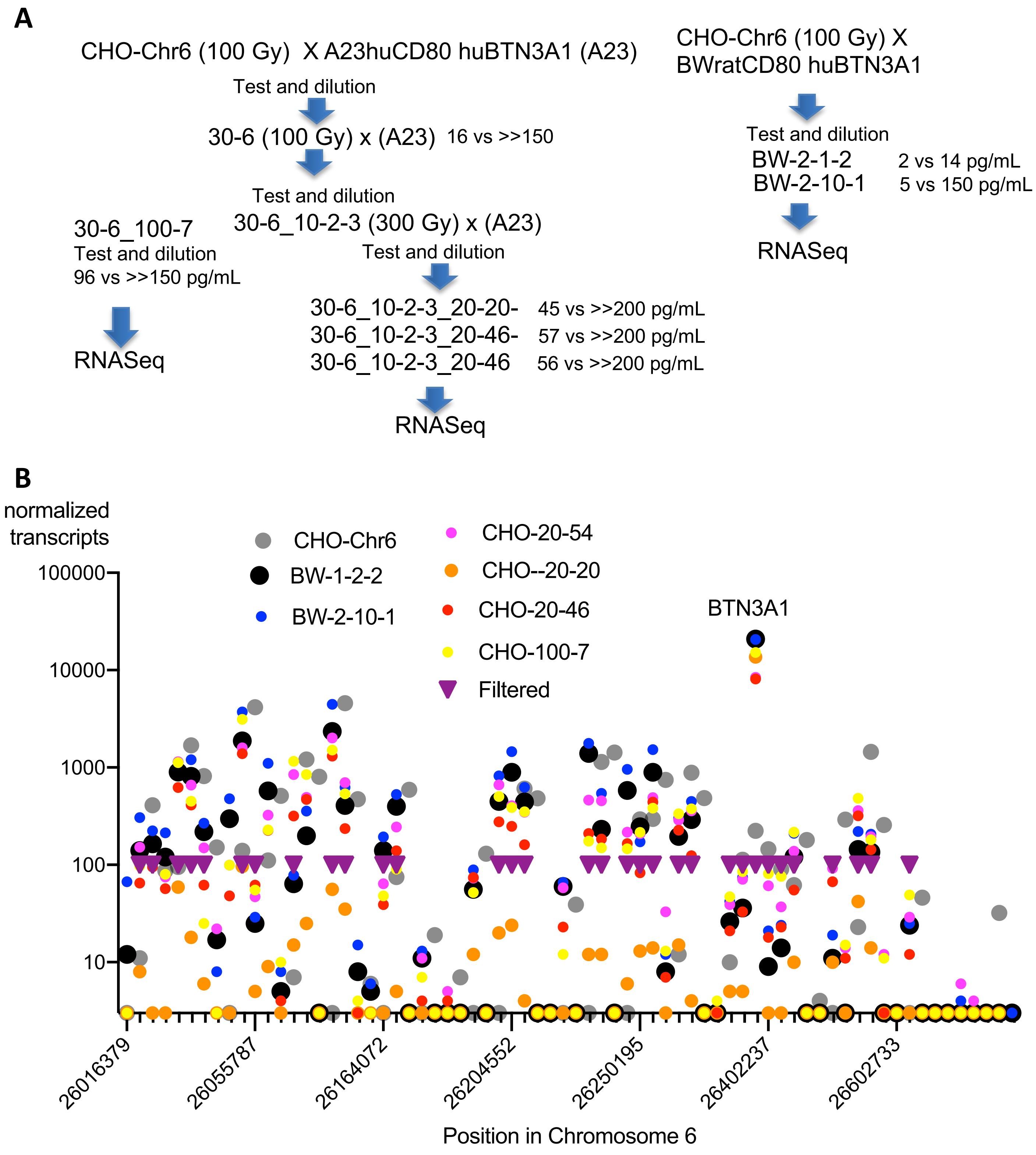

Supplementary Figure 1, related to Figure 1. Strategy used for identification of BTN2A1 as Factor X. (A) Origin of radiation hybrid clones selected for transcriptional analysis. IL-2 production (pg/ml) in the stimulation assay in the presence of medium or with 1 µM HMBPP. (B) Region of human Chr 6 implicated in potentiation of P-Ag sensitisation by rodent-human radiation hybrids. Each dot represents a gene detected as ≥3 transcripts, with those highlighted in pink detected in every P-Ag-sensitizeable clone selected for analysis.

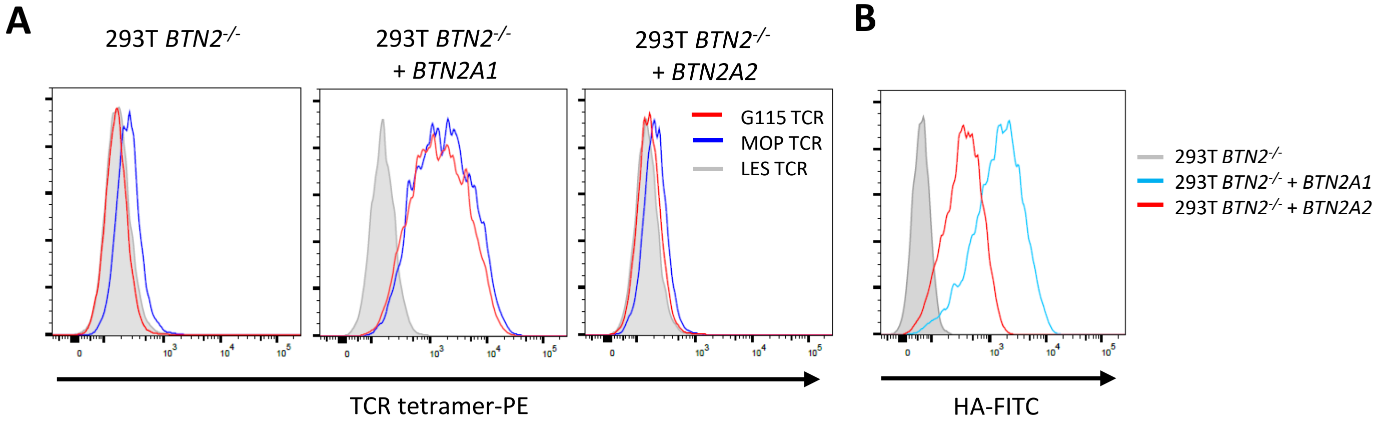

Supplementary Figure 2, related to Figure 2. Vγ9Vδ2 TCR tetramer stains BTN2A1 transductants. (A) 293T *BTN2*$^{-/-}$ cells transduced to express HA-tagged BTN2A1 and BTN2A2 were stained with the indicated TCR tetramers. (B) Flow cytometry analysis of HA-tagged BTN2A1 and BTN2A2 in 293T *BTN2*$^{-/-}$ cells, as detected by anti-HA mAb.

Supplementary Figure 2

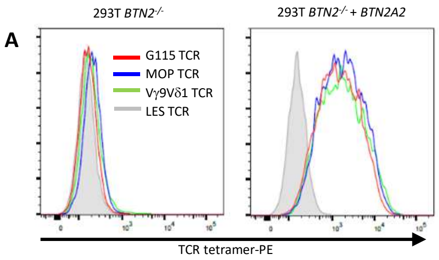

TCR clone	Vγ	Jγ	CDR3γ	Vδ	Jδ1	CDR3δ
MOP	Vγ9	JγP	CALKELGKKIKVF	Vδ2	Jδ1	CACDPVVLGDTGYTDKLIF
G115	Vγ9	JγP	CALWEAQQELGKKIKVF	Vδ2	Jδ1	CACDTLGMGGEYTDKLIF
D1C55	Vγ9	JγP	CALWEWVTQELGKKIKVF	Vδ2	Jδ1	CACDTVLGDRSRTDKLIF
Vγ9Vδ1	Vγ9	Jγ2	CALWEVQSVEKLF	Vδ1	Jδ1	CALGDLTGVPITDKLIF
Vγ2Vδ1	Vγ2	Jγ2	CATWDGPLNYKKLF	Vδ1	Jδ1	CALGEKNPVRNWGYNNDKLIF
LES	Vγ4	Jγ1	CATWDGFYYKKLF	Vδ5	Jδ1	CAASSPIRGYTGSDKLIF

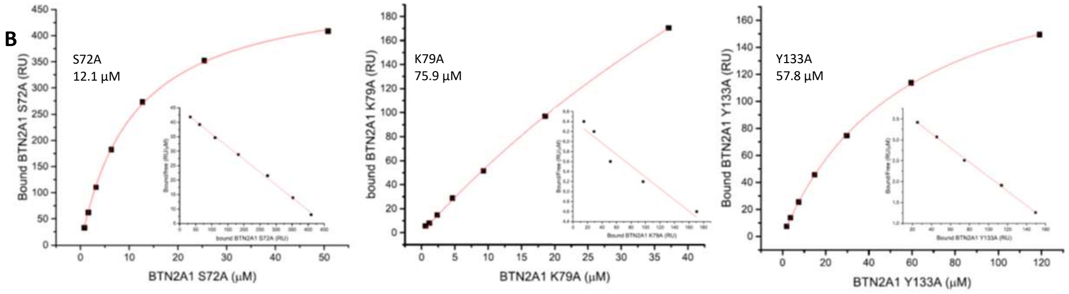

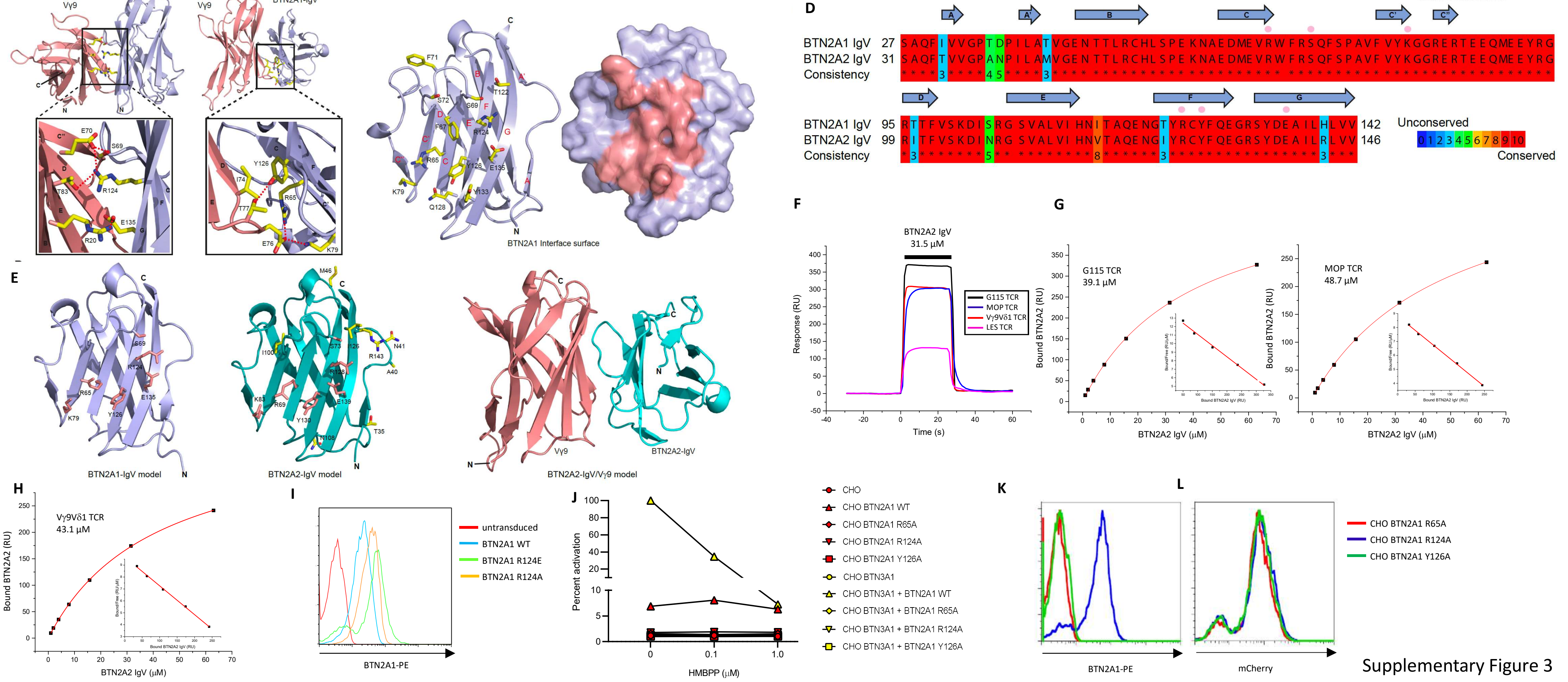

Supplementary Figure 3

Supplementary Figure 3, related to Figure 3. BTN2A1 and BTN2A2 bind germline encoded regions of Vγ9. (A) (Left panel) Vγ9Vδ1-TCR tetramer staining of BTN2A1-transduced 293T *BTN2*$^{-/-}$ cells. (Right panel) Comparison of CDR3γ sequences in G115, MOP, D1C55, and Vγ9Vδ1 TCR clonotypes. (B) Equilibrium binding of BTN2A1 mutants S72A (left; 12.1 μM)), K79A (middle, 75.9 μM) and Y133A (right, 57.8 μM) to G115 TCR. Results are representative of two independent experiments. (C) (Left two panels) Vγ9/BTN2A1 interface from the mutagenesis-informed HADDOCK model of the interaction. (Right two panels) BTN2A1 interface residues (left) forming a surface (right) proposed to interact with Vγ9 IgV. (D) Comparison of BTN2A2 and BTN2A1 IgV sequence. Pink dots indicate conservation of BTN2A1 IgV amino acids predicted to be involved in binding Vγ9 IgV, in BTN2A2. (E) Homology model of BTN2A2 IgV, alone and in complex with Vγ9 TCR. (F) Specific binding of BTN2A2 IgV (31.5 μM) to Vγ9Vδ2 TCR as detected by SPR. (G) Equilibrium affinity and Scatchard (inset) analysis of BTN2A2 IgV binding to G115 (39.1 μM) and MOP (48.7 μM) Vγ9Vδ2 TCRs. (H) Equilibrium affinity analysis of BTN2A2 IgV binding to Vγ9Vδ1 TCR (43.1 μM). (I) Flow cytometry staining of BTN2A1 R124A and BTN2A1 R124E mutants at surface of BTN3A1-expressing CD80^{+} CHO cells. (J) Effects of BTN2A1 mutations on IL-2 production by TCR-MOP in response to HMBPP-treated BTN3A1 and BTN2A1 expressing CD80^{+} CHO cells. (K) Flow cytometry staining of BTN2A1 R65A, R124A and Y126A mutants at surface of BTN3A1-expressing CD80^{+} CHO cells. (L) mCherry reporter detection in BTN2A1 R65A, R124A and Y126A mutants-expressing CD80^{+} CHO cells.

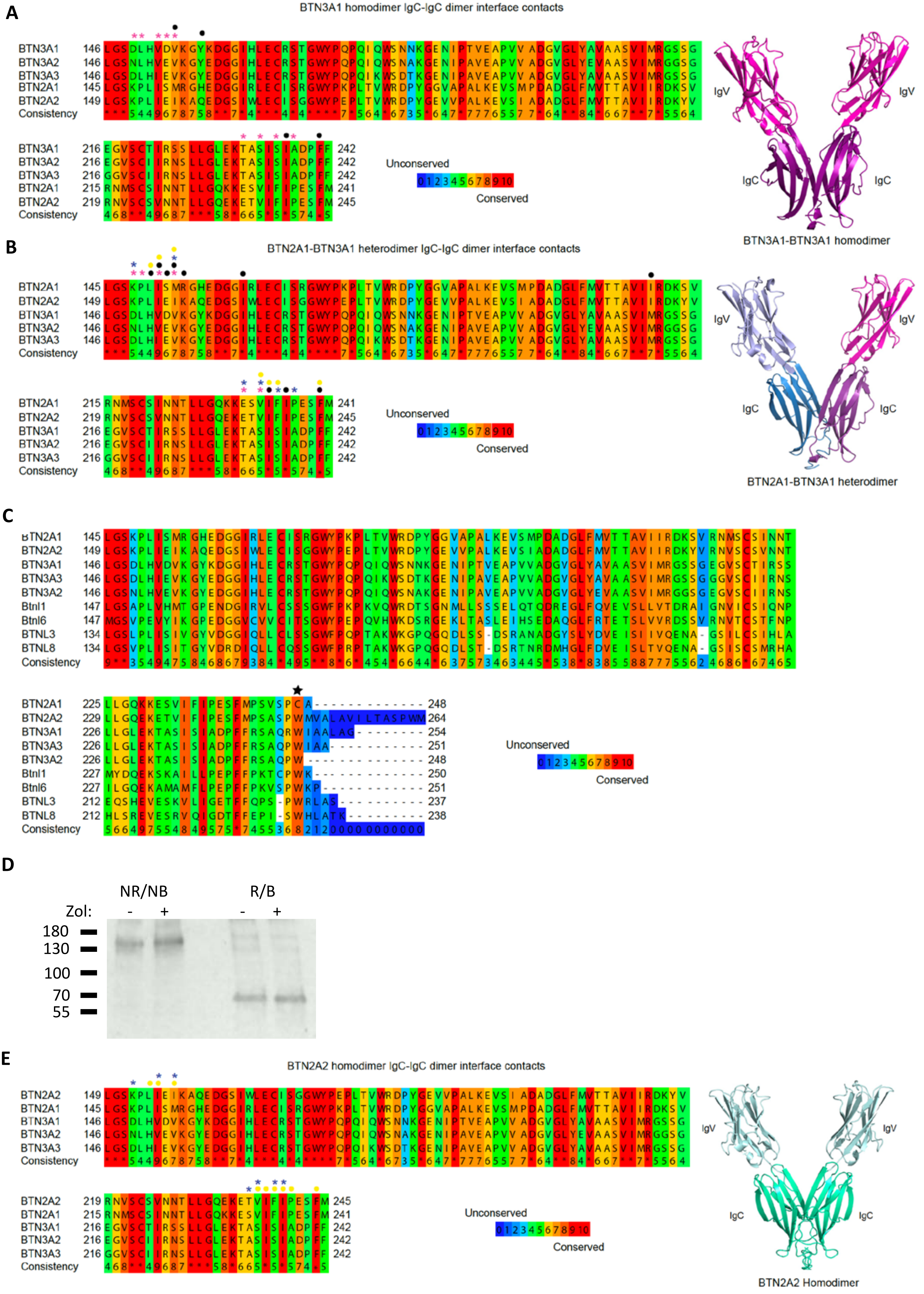

Supplementary Figure 4

Supplementary Figure 4, related to Figure 4. BTN2A1 can form a homodimer involving hydrophobic IgC-IgC contacts and a disulphide linkage. (A) Sequence alignment showing conservation of BTN3A1 IgC-IgC interface residues in BTN2A1. Pink stars indicate BTN3A1 hydrogen bonding interactions mediated by main chain atoms (D149, L150, V152, D153, V154, T232, S234, S236 and A238); black spheres indicate BTN3A1 hydrophobic interactions (V154, Y157, I237, and F241). (B) Sequence alignment showing potential for heterodimer interactions involving BTN2A1. Pink stars indicate BTN3A1 hydrogen bonding interactions mediated by main chain atoms (D149, L150, V152, V154, T232 and S234); black spheres indicate BTN3A1 hydrophobic interactions (H151, V152, D153, V154, K155, I162, M210, I235, I237 and F241); blue stars indicate BTN2A1 hydrogen bonding interactions mediated by main chain atoms (K148, M153, E231, V233, F235 and P237); yellow spheres indicate BTN2A1 hydrophobic interactions (L150, I151, M153, V233, I234, F235 and F240). (C) Sequence alignment showing unique membrane proximal Cys residue (highlighted by a black star) in BTN2A1 relative to other BTN/BTNL molecules. (D) BTN2A1 homodimer formation is not affected by Zol treatment. $CD80^{+}$ CHO cells expressing BTN2A1-HA and FLAG-BTN3A1 were treated +/- Zol (20 μM) overnight, surface biotinylated, then lysed and immunoprecipitated using anti-HA antibody, and blotted with streptavidin-horseradish peroxidase. (E) BTN2A2 dimer formation. Homology model of BTN2A2 indicating potential for IgC-IgC homodimers. Blue stars indicate BTN2A2 hydrogen bonding interactions mediated by main chain atoms (K152, I155, I157, T236, V237, F239 and I240); yellow spheres indicate BTN2A2 hydrophobic interactions (L154, I155, I157, V237, I238, F239, I240, P241 and F244).

Supplementary Figure 5

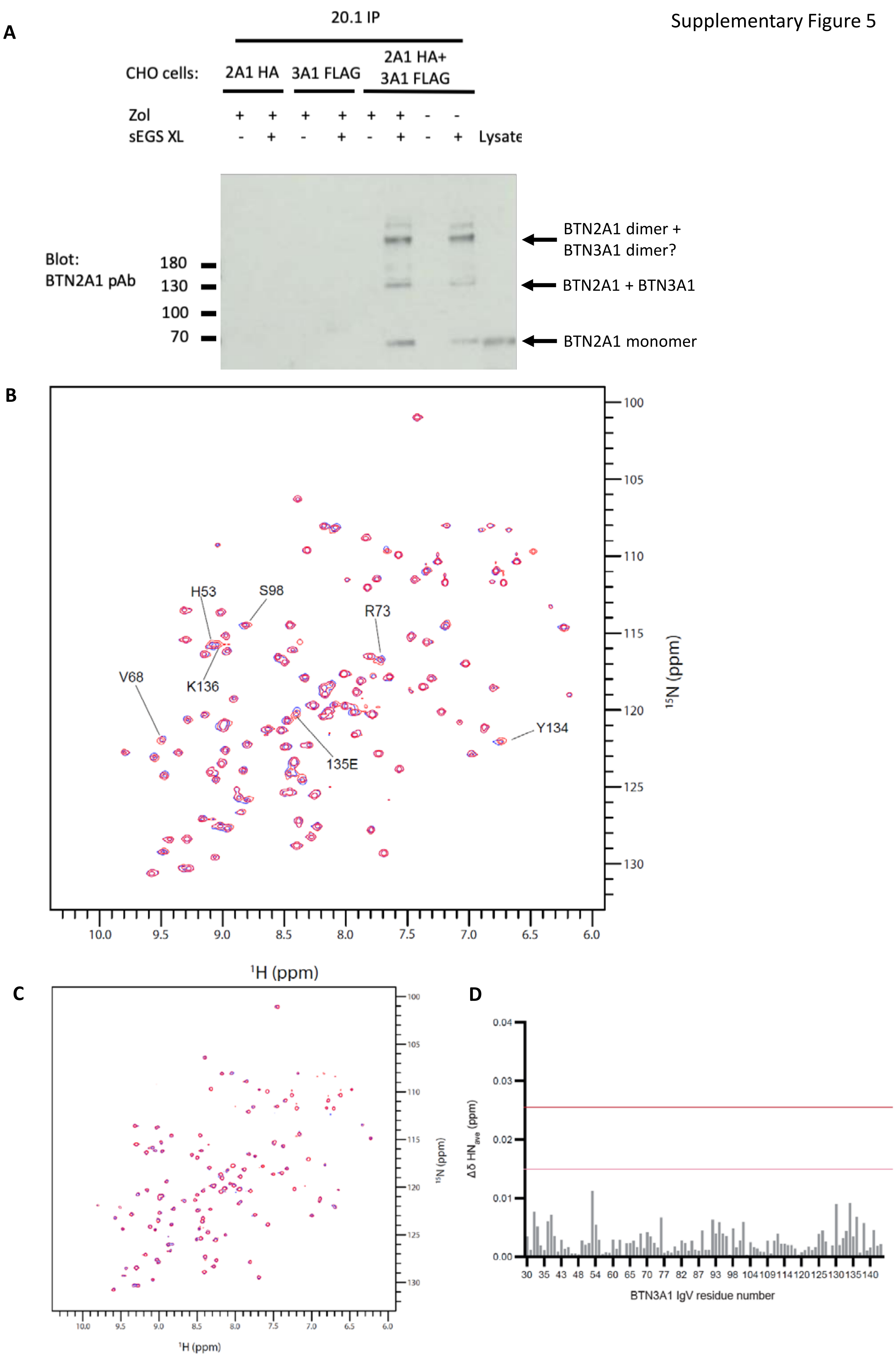

Supplementary Figure 5, related to Figure 5. BTN2A1 associates with BTN3A1 via IgV-IgV contacts. (A) SDS-PAGE following surface crosslinking and lysis of CHO CD80^{+} cells transduced with BTN2A1-HA and FLAG-BTN3A1, and subsequent immunoprecipitation using 20.1 anti-BTN3A1 mAb, followed by western blot using anti-BTN2A1 polyclonal antibody. (B) NMR chemical shift perturbation analysis of BTN3A1 IgV domain (100 µM) with BTN2A1 IgV. Overlay of ^{1}H-^{15}N HSQC spectra of ^{1}H-^{15}N-labelled BTN3A1 IgV domain in the absence (red) and presence (100 µM; blue) of BTN2A1 IgV domain. Labelled residues are those undergoing CSPs above the threshold set in Figure 5D. (C) NMR chemical shift perturbation analysis of BTN3A1 IgV domain (120 µM) with BTNL3 IgV. Overlay of ^{1}H-^{15}N HSQC spectra of ^{1}H-^{15}N-labelled BTN3A1 IgV domain in the absence (red) and presence (320 µM; blue) of BTNL3 IgV domain. (D) Graph of chemical shift versus residue number in BTN3A1 IgV following addition of BTNL3 IgV domain as in (C). Threshold levels for significant CSPs are indicated by horizontal lines as in Figure 5D.

Supplementary Table 1, related to main text Figure 1 and Key Resource Table. Primers and genomic markers used for characterization of radiation hybrids.

Marker	Fwd primer	Rev primer	Source	Identifier
Ly86	AAATCTTCTTCCCCTGGGAA	GAGGATGGCTGTTAGATCATCC	This Paper	N/A
CD83	TAAGGGAAAGCCAACAATGG	CACGGTCTGTTCTTGAAGCA	This Paper	N/A
RN7SL	GAATGTGGATGGGCTGTCTC	GCCTCAAACAGTGCATTTATACTT	This Paper	N/A
MYLIP	CAGACATGCAGAAAGTTGCA	ATGCACACAGAAGATAAAGCA	This Paper	N/A
NUP153	GGACTCTCAGGCAGGAAT	TGGAACAGCCCAATAGGT	This Paper	N/A
RPL21	CCGCGGAATTCTTAGTTATCCTCT	TTTTTGCAAAGCTCACACCTTCT	This Paper	N/A
RPL36	TGTGTAGCGGAAGACATCTAGCA	TCCATGAGCACTGGTAAGAATGA	This Paper	N/A
Prolactin	GGTTTCTGATACACTGGCCCGATAT	GGAACGGATCATTAAAGGACCTTCTC	Cathy Abbot	N/A
LOC1053	TCAGACCAAGAACAACGAGGAAC	ACTCAACACGTGTCACACACAGA	This Paper	N/A
ACOT13	GTATTGTAACAGGGGTCATGCA	CCCCAAGCTGAGACATGATT	This Paper	N/A
C6orf229	GATCCTGCTGCTCCAGAGTG	AAGCTCCTTGACGCAAGACA	This Paper	N/A
MTCO2P33	CAATTTGGCCTTCAAACGCT	AACACGTAAGGATAGGAGGGC	This Paper	N/A
RP3-425P12	AGATCATTTCCTGAAGGCCGA	CCTTGCATTCTTTGGCGGGT	This Paper	N/A
SLC17A4	AGGCTGAGGCATCCCTA	TGTACCCCGGCTAATAAGTT	This Paper	N/A
BTN3A2	AGCACTTTACTGATATTCATTC	AAAGTCAATACACTTTTCTACAC	This Paper	N/A
OR2B2	AATTTAAAATCACAGTGAGTAG	AGATGGAGGTTCAGATAG	This Paper	N/A
MOG	AACTGAAAGGGTTCCGTGTG	ATTTCTGACCATTTGCCCTG	This Paper	N/A
TNFA	CAGGGTCCTACACACAAATCAGTCA	AAGAGAACCTGCCTGGCAGCTTGT	This Paper	N/A
HLA-DQ	GGTTATCACTCTTCTGTGATGCC	TCTGGGTCAGTGCAGGAAG	This Paper	N/A
STK38	TTGCTACTCAGCCTATTACTTTT	AAGGTTATACAGGACGACATAAC	This Paper	N/A
NFKBIE	GCCTTTTACTTCAACTGCCCTCT	ACAACAGGTGCCAGTTACAGGTT	This Paper	N/A
ICK	ACAAAAAAGCCTACTGCATTCA	CTGTCTGCCTTGGTGATGAATC	This Paper	N/A
EEF1A1	CCAACCTGAGATGTCCCTGT	TGAGTCACCCACACAAAGGA	This Paper	N/A
MAP3K7	TTGCTTAACCTTTTCCATTTCC	TCTGAATGTCACAGGCTGTACC	This Paper	N/A
CD164	GAGGCACCCCATAACACTTG	TGAATTTTGGAGATGGAATGC	This Paper	N/A
PTPRK	CCACCATTATTCAATTCCAAGCAT	TGGTCTCTTAATGCCTGATTGGT	This Paper	N/A
MYB	ACAAGCATGCGTTGCCACTTCTT	CAAACACAGGATCCATGCAACA	This Paper	N/A
ULBP2	CATCAAAATGCGCATTCATC	CCAGATCAGAATGGGTGCTT	This Paper	N/A
ESR	CTCTCTCTCTGCGCATTCAG	GAAGCCCAGAGATGCCTCAC	Cathy and S	N/A
SCAF8	TGGCACTAAATGAGGAAAAA	TGCCTTCATGGAGTTTACAT	This Paper	N/A
MAS1	CGGTCACAGTTGAGACTGTCGTCT	TTAGTATCTCATGCATATGGGATGAG	Theune et a	N/A
SLC44A4	GTCCTCTCCCACAAAAAAGTCC	TCAGCCTTCCCCAGTTCC	This Paper	N/A
TRAM2	CTTATTTGGTGCCATGCAATG	AGGGTGAGAAGGACCTTTAAGG	This Paper	N/A
GPCR6	CGTGTCCTCTCACCAACACC	AGCTCCACATCCTGAACACC	This Paper	N/A
ARG1	GTACTATGTGTCCATGTCATTC	TTAGTAGTTTTAAGCAGGACGTT	This Paper	N/A

Supplementary Table 2, related to Key Resource Table and STAR methods. Cloning and CRISPR primers.

Reagent or Resource	Source	Identifier
BTN2A-IgVgeno CRISPR Fwd (GCAGTGTTTGTGTATAAAGG)	This Paper	N/A
BTN2A-IgVgeno CRISPR Rev (CCTTTATACACAAACACTGC)	This Paper	N/A
BTN2A1geno 49FCRISPR Fwd (AATGAGGGGCCATGAAGACG)	This Paper	N/A
BTN2A1geno 49FCRISPR Rev (CGTCTTCATGGCCCCTCATT)	This Paper	N/A
BTN2A2geno 343CRISPR Fwd (TGAGGCCATCCTACGCCTCG)	This Paper	N/A
BTN2A2geno 343CRISPR Rev (CGAGGCGTAGGATGGCCTCA)	This Paper	N/A
pMIM-BTN2A1Fwd (CTTCTCTAGGCGCCGGAATTCGCCGCCACCATGGAATCAGCTGCTGCC)	This Paper	N/A
pMIM-BTN2A1Rev (TCCGCTAGCTACGTAAGATCTCTATAGGCTCTGGTGACC)	This Paper	N/A
pMIG-BTN2A2Fwd (AAATGAATTCGCCGCCACCATGGAACCAGCTGCTGCTC)	This Paper	N/A
pMIG-BTN2A2Rev (AAATAGATCTCTATAGGCTCTGGTGGGTCC)	This Paper	N/A
BTN2A1IgV Geno Fwd (CCCTTTGTTGAACAGCCCAG)	This Paper	N/A
BTN2A1IgV Geno Rev (CCAAAGTCACACTGTGCCAA)	This Paper	N/A
BTN2A2IgV Geno Fwd (CTGTAGCCTTGGAATATCTGCTTC)	This Paper	N/A
BTN2A2IgV Geno Rev (CTTTGTGCAAGAGGAAGCCACTTT)	This Paper	N/A
BTN2A1 49F Geno Fwd (ATGGGGCCTATAGGTGGACCG)	This Paper	N/A
BTN2A1 49F Geno Rev (AGGGTGTTGTTGATAGAGCAGGACATG)	This Paper	N/A
BTN2A2 343 Geno Fwd (AATATCTGCTTCCTTTCATCCCTG)	This Paper	N/A
BTN2A2 343 Geno Rev (GTGAAACCTATAGCTTAGCAGGGC)	This Paper	N/A
BTN2A1 R65A Fwd (AGGACATGGAGGTGGCGTGGTTCCGGTCTC)	This Paper	N/A
BTN2A1 R65A Rev (GAGACCGGAACCACGCCACCTCCATGTCCT)	This Paper	N/A
BTN2A1 R124A Fwd (AAAACGGCACCTACGCCTGTTACTTCCAAG)	This Paper	N/A
BTN2A1 R124A Rev (CTTGGAAGTAACAGGCGTAGGTGCCGTTTT)	This Paper	N/A
BTN2A1 Y126A Fwd (GCACCTACCGCTGTGCCTTCCAAGAAGGCA)	This Paper	N/A
BTN2A1 Y126A Rev (TGCCTTCTTGGAAGGCACAGCGGTAGGTGC)	This Paper	N/A
BTN2A1 R124E Fwd (CACCTACGAATGTTACTTCCAAGAAGGCAGG)	This Paper	N/A
BTN2A1 R124E Rev (GGAAGTAACATTCGTAGGTGCCGTTTTCC)	This Paper	N/A
BTN2A1 C247W Fwd (TGTGTCTCCCTGGGCAGTGGCCCTGCCTATC)	This Paper	N/A
BTN2A1 C247W Rev (GGGCCACTGCCCAGGGAGACACACTGGGC)	This Paper	N/A
pIZ-BTN2A1 Fwd (GACCATCCTCTAGAGAATTCGCCGCCACCATGGAATCAGCT)	This Paper	N/A
pIZ-BTN2A1-HA Rev (GGGAGGGAGAGGGGGATCCCTAAGCGTAATCTGGAACATCGTATGGGTATGCTGATCCTAGGCTCTGGTGGGTCCCCACT)	This Paper	N/A
pIZ-BTN2A2 Fwd (GACCATCCTCTAGAGAATTCGCCGCCACCATGGAACCAG)	This Paper	N/A
pIZ-BTN2A2-HA Rev (GGGAGGGAGAGGGGGATCCCTAAGCGTAATCTGGAACATCGTATGGGTATGCTGATCCTAGGCTCTGGTGGGTCCCCACTC)	This Paper	N/A
pIZ-BTN2A1Ldr-HA Fwd (ATGCCaaTTGGCCACCATGGAATCAGCTGCTGCCCTGCACTTCTCCCGGCCAGCCTCCCTCCTCCTCCTCCTCAGCCTGTGTGCACTGGTCTCAGGATCAGCATACCCATACGA)	This Paper	N/A
pIZ-BTN2A1Ldr-HA Rev (TGTGTGCACTGGTCTCAGGATCAGCATACCCATACGATGTTCCAGATTACGCTGCTGGATCCACCATGGTAATTGAATTCAATATTTAAATAATAGATct)	This Paper	N/A
pIZ-HA-BTN2A1 Fwd (CCAGATTACGCTGCTGGATCCGCCCAGTTTATTGTCGTGG)	This Paper	N/A
pIZ-BTN2A1 Rev (TATTATTTAAATATTGAATTCCTATAGGCTCTGGTGGGTCC)	This Paper	N/A
pIZ-HA-BTN2A2 Fwd (CCAGATTACGCTGCTGGATCCGCCCAGTTTACTGTCGTGG)	This Paper	N/A
pIZ-BTN2A2-HA Rev (TATTATTTAAATATTGAATTCCTATAGGCTCTGGTGGGTCCC)	This Paper	N/A
pIH-BTN3A1Ldr-FLAG Fwd (GGATCAGCACAATTGGCCACCATGAAAATGGCAAGTTTCCTGGCCTTCCTTCTGCTCAACTTTCGTGTCTGCCTCCTTTTGCTTCAGCTGCTCATGCCTCACTCAGCTGGATCAGCAGACTACAA)	This Paper	N/A
pIH-BTN3A1Ldr-FLAG-Rev (TCCTGATGCAGATCTATTATTTAAATATTGAATTCAATTACCATGGTGGATCCAGCCTTGTCATCGTCGTCCTTGTAGTCTGCTGATCC)	This Paper	N/A
pIH-FLAG-BTN3A1 Fwd (CAAGGCTAGATCTCAGTTTTCTGTGCTTGGAC)	This Paper	N/A
pIH-BTN3A1 Rev (TATTGAATTCTCACGCTGGACAAATAGTCAG)	This Paper	N/A
MOP Vγ9 Fwd (GGACCATCCTCTAGAGAATTCGCCGCCACCATGCTGTCACTGCTGCTCC)	This Paper	N/A
MOP Vγ9 Rev (GGGGGAGGGAGAGGGGGATCCTTATGATTTCTCTCCATTGCAGCAG)	This Paper	N/A
MOP Vγ9-E70A Fwd (CAAATTTGCGGTGGATAGGATACCTGAAACGTC)	This Paper	N/A
MOP Vγ9-E70A Rev (TCCTATCCACCGCAAATTTGCCTGACGGAATG)	This Paper	N/A
MOP Vγ9-E70R Fwd (CAAATTTAGGGTGGATAGGATACCTGAAACGTC)	This Paper	N/A
MOP Vγ9-E70R Rev (TCCTATCCACCCTAAATTTGCCTGACGGAATG)	This Paper	N/A
MOP Vγ9-E70K Fwd (CAAATTTAAGGTGGATAGGATACCTGAAACGTC)	This Paper	N/A
MOP Vγ9-E70K Rev (TCCTATCCACCTTAAATTTGCCTGACGGAATG)	This Paper	N/A
